# Control of Gastric H,K-ATPase Activity by Cations, Voltage and Intracellular pH Analyzed by Voltage Clamp Fluorometry in *Xenopus* Oocytes

**DOI:** 10.1371/journal.pone.0033645

**Published:** 2012-03-20

**Authors:** Katharina L. Dürr, Neslihan N. Tavraz, Thomas Friedrich

**Affiliations:** Institute of Chemistry, Technical University of Berlin, Berlin, Germany; University of Cambridge, United Kingdom

## Abstract

Whereas electrogenic partial reactions of the Na,K-ATPase have been studied in depth, much less is known about the influence of the membrane potential on the electroneutrally operating gastric H,K-ATPase. In this work, we investigated site-specifically fluorescence-labeled H,K-ATPase expressed in *Xenopus* oocytes by voltage clamp fluorometry to monitor the voltage-dependent distribution between E_1_P and E_2_P states and measured Rb^+^ uptake under various ionic and pH conditions. The steady-state E_1_P/E_2_P distribution, as indicated by the voltage-dependent fluorescence amplitudes and the Rb^+^ uptake activity were highly sensitive to small changes in intracellular pH, whereas even large extracellular pH changes affected neither the E_1_P/E_2_P distribution nor transport activity. Notably, intracellular acidification by approximately 0.5 pH units shifted V_0.5_, the voltage, at which the E_1_P/E_2_P ratio is 50∶50, by −100 mV. This was paralleled by an approximately two-fold acceleration of the forward rate constant of the E_1_P→E_2_P transition and a similar increase in the rate of steady-state cation transport. The temperature dependence of Rb^+^ uptake yielded an activation energy of ∼90 kJ/mol, suggesting that ion transport is rate-limited by a major conformational transition. The pronounced sensitivity towards intracellular pH suggests that proton uptake from the cytoplasmic side controls the level of phosphoenzyme entering the E_1_P→E_2_P conformational transition, thus limiting ion transport of the gastric H,K-ATPase. These findings highlight the significance of cellular mechanisms contributing to increased proton availability in the cytoplasm of gastric parietal cells. Furthermore, we show that extracellular Na^+^ profoundly alters the voltage-dependent E_1_P/E_2_P distribution indicating that Na^+^ ions can act as surrogates for protons regarding the E_2_P→E_1_P transition. The complexity of the intra- and extracellular cation effects can be rationalized by a kinetic model suggesting that cations reach the binding sites through a rather high-field intra- and a rather low-field extracellular access channel, with fractional electrical distances of ∼0.5 and ∼0.2, respectively.

## Introduction

**Figure 1:**
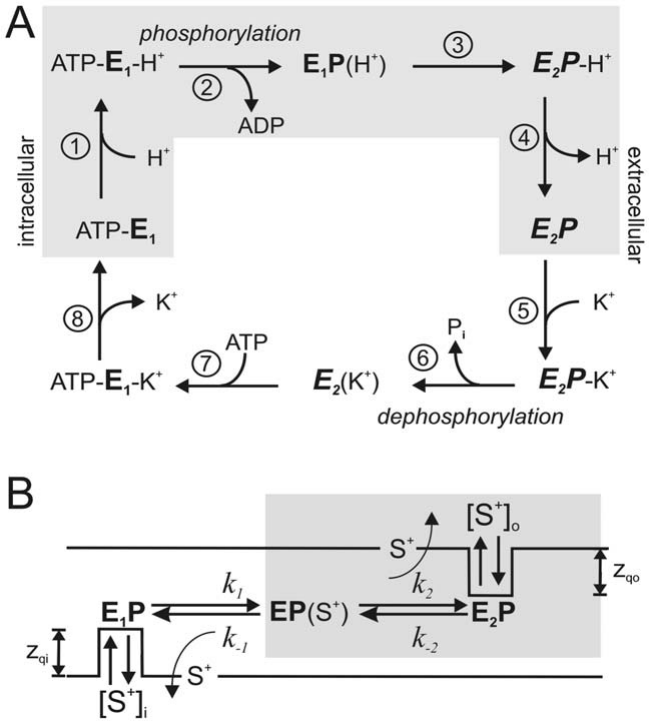
Reaction cycle of gastric H,K-ATPase. (A) Reaction mechanism of gastric H,K-ATPase adapted from the Post-Albers scheme [1], [2], which had originally been postulated for the related Na,K-ATPase. Upon intracellular binding of protons to the E_1_ conformation (step 1), a phosphointermediate with occluded H^+^ ions (E_1_P(H^+^)) is formed (step 2), and after a conformational change to E_2_P (step 3), protons dissociate to the extracellular space (step 4). Subsequently, K^+^ ions bind from the extracellular side (step 5) and become occluded, a process which stimulates dephosphorylation (step 6), and after a conformational change from E_2_ to E_1_ (step 7) the K^+^ ions are intracellularly released (step 8). The gray box indicates the reaction sequence which can be studied by voltage pulses at [K^+^]_ext_ = 0 in VCF experiments. (B) Pseudo three-state model for the reaction sequence including steps 1 to 4 in (A). A detailed description and analysis of this kinetic scheme is provided in Supporting Information (**Appendix S2**).

Gastric H,K-ATPase, the main transporter responsible for acid secretion in the stomach, belongs to the family of P-type ATPases. A hallmark of this ATPase family is the formation of phosphorylated enzyme intermediates during the transport cycle, which is achieved by reversible phosphorylation of a highly conserved aspartate residue (Asp-385 in rat gastric H,K-ATPase). The phosphorylation and dephosphorylation reactions are coupled to conformational transitions between the two principal conformations E_1_ and E_2_ (and the respective phosphoenzyme forms E_1_P and E_2_P, respectively), for which the ion binding pocket is exposed to different sides of the membrane. Furthermore, the conformational changes are linked to characteristic changes in the affinities for the transported cations. For the reaction mechanism of Na,K-ATPase, a cyclic scheme of reversible partial reactions has been proposed, which is known as Post-Albers scheme [1], [2]. Although Na,K-ATPase exchanges 3 Na^+^ against 2 K^+^ ions in an overall electrogenic transport reaction, which is in contrast to the 2∶2 (or 1∶1), hence electroneutral, H^+^/K^+^ exchange mediated by the gastric H,K-ATPase, the pump cycle of gastric H,K-ATPase presumably proceeds according to a very similar reaction scheme (Fig. 1A).

Voltage-dependent partial reactions of the Na,K-ATPase have been studied in detail. It is well established that the major electrogenic event in the Na,K-ATPase cycle occurs during extracellular Na^+^ release (or re-uptake). This has first been inferred from the voltage-dependent inhibition of K^+^-stimulated stationary pump currents by extracellular Na^+^ [3], [4], [5]. Furthermore,ouabain-sensitive presteady-state currents, which occur in the absence of extracellular K^+^ in response to voltage pulses under conditions favoring phosphoenzyme formation (intracellular Na^+^ and ATP present), critically depend on the presence of extracellular Na^+^ [6], [7], [8]. These findings were interpreted in terms of a high-field ion access channel or “ion well” [9], [10], [11] through which the Na^+^ ions travel upon extracellular release from the binding sites. Since the E_1_P↔E_2_P transition is rate-limiting for Na^+^ deocclusion/release as well as Na^+^ uptake/occlusion, the voltage dependence of the major charge component of the transient currents represents the voltage-dependent E_1_P/E_2_P distribution according to a Boltzmann-type function, which is strongly dependent on [Na^+^]_ex_. Its *V_0.5_* value, the membrane voltage at which the E_1_P/E_2_P ratio is 50∶50, shifts to positive potentials with increasing [Na^+^]_ex_ implying that less hyperpolarizing potentials are required to drive the distribution towards E_1_P [12]. Further studies on giant squid axons with improved time resolution revealed three distinct and sequential phases in the presteady-state charge movements reflecting the strictly sequential deocclusion and release of the three Na^+^ ions [13]. Importantly, the lower electrogenicity observed for the release of the second and the third Na^+^ ion (apparent fractional electric distance: ∼0.25) compared to the “first” Na^+^ ion (∼0.7–0.8) suggests that the ion access channel is significantly restructured upon release of the first Na^+^ ion, yielding a rather shallow ion well for the remaining two. The reduced electrogenicity of the second and third Na^+^ is matched by a similarly low one for the subsequent binding of extracellular K^+^ [14]. Other, albeit weaker, electrogenic steps in the Na,K-ATPase pump cycle have been attributed to intracellular Na^+^ binding [15], [16] and the E_1_P-E_2_P conformational change [17], [18].

Due to its overall electroneutral transport, much less is known about the voltage-dependent steps of gastric H,K-ATPase. Experiments in which H,K-ATPase-containing parietal cell membrane fragments were adsorbed to black lipid membranes [19], [20], [21] provided evidence for electrogenicity in the H^+^ limb of the transport cycle, since rapid release of ATP from caged ATP in the absence of K^+^ induced transient currents. To account for the overall electroneutrality, it was proposed that electrogenic H^+^ translocation is counter-balanced by another partial reaction of opposite electrogenicity during K^+^ translocation. Indeed, K^+^ inhibition experiments on inside-out gastric vesicles [22] revealed that an electrogenic step exists in the K^+^ branch (steps 5–8 in Fig. 1A). These studies showed that the inhibitory effect of high intracellular [K^+^] on ATPase activity was prevented at intracellularly negative K^+^ diffusion potentials, but was restored upon dissipation of the diffusion potential. Moreover, equilibrium titration experiments using the electrochromic dye RH421 on gastric membrane vesicles confirmed the electrogenicity of both K^+^ and H^+^ binding steps [23].

Several studies have shown that Na^+^ modulates function of the gastric H,K-ATPase. However, interpretation of the results was hampered because the used vesicle or membrane preparations did not allow a differentiation between intra- and extracellular effects [24], [25], [26], [27]. We have previously found in Rb^+^ uptake experiments using *Xenopus* oocytes that extracellular Na^+^ reduces the apparent affinity for Rb^+^ about 7-fold, thus indicating a competition between Na^+^ and Rb^+^ [28]. This behavior is quite similar to the Na,K-ATPase, which exhibits significantly decreased apparent K^+^ affinity in the presence of extracellular Na^+^ as well [14]. In order to understand the function of H,K-ATPase within its physiological context, it is mandatory to study the complexity of extra- and intracellular cation effects and their voltage dependence in intact cells.

A suitable technique for this purpose is voltage clamp fluorometry (VCF), which senses voltage-dependent partial reactions even in transporters that operate net electroneutrally. Initially, VCF has been pioneered for the detection of conformational rearrangements of voltage sensing segments in voltage-gated cation channels [29], [30]. To enable SH-reactive coupling of fluorescent dyes for site-specific labeling, cysteine mutations are introduced into extracellular loops of the protein, usually at the interface between the membrane and the extracellular space. Here, conformational transitions may change in the dye's microenvironment and induce variations in fluorescence intensity, which, depending on the photophysical properties, can be due to local changes in the electrostatics, hydrophobicity, pH, or differential access of fluorescence quenchers. Tetramethylrhodamine-maleimide (TMRM) has proven distinctly useful, since it is particularly sensitive to solvent polarity and collisional quenching by water [29]. Thus, its fluorescence increases upon movement into a sheltered, hydrophobic environment, and quenching occurs upon exposure to the aqueous phase. When TMRM was used to label the Shaker K^+^ channel (mutation M356C at the N-terminal part of the S4 segment, which carries most of the gating charge), a good kinetic correlation between fluorescence changes and the gating charge integral was found. Other dyes like fluoresceine-maleimide or Oregon Green maleimide attached to the same residue produced kinetically more complex responses [29] indicating that these fluorophores encounter a series of different microenvironments, which are sometimes difficult to correlate with functional properties. For the Na,K-ATPase, it has been shown that TMRM labeling close to the extracellular end of the central M5 helix (mutation N790C, sheep α_1_-subunit), leads to fluorescence signals with properties similar to those of presteady-state currents [31] indicating that the same molecular event, the E_1_P↔E_2_P transition, is reported. For H,K-ATPase, TMRM labeling at homologous position (mutation S806C, Figure S2), produces similar fluorescence signals upon voltage pulses in the absence of extracellular K^+^, which presumably reflect the E_1_P↔E_2_P relaxation as well [28], [32], [33], [34].

In the present study, we used the VCF technique and carried out Rb^+^ uptake measurements under various pH and ionic conditions upon expression of gastric H,K-ATPase in *Xenopus* oocytes. This dual approach allowed us to gain information about the voltage dependence of the overall pump process as well as that of a subset of partial reactions involving the E_1_P↔E_2_P conformational change and the ion translocation steps linked to it. Our data show that the E_1_P/E_2_P distribution and stationary cation transport are rather insensitive to extracellular pH changes, but tightly regulated by intracellular pH, and that pump turnover is rate-limited by a partial reaction step early in the H^+^ limb of the cycle.

## Materials and Methods

### Ethics statement

Surgical removal of ovary tissue from adult *Xenopus laevis* females followed registered protocols approved by the relevant state authority (Landesamt für Gesundheit und Soziales Berlin, Reg. No. O 0308/06) and the local ethics committee (Tierschutzbeirat), in strict accordance with the German Animal Protection Act (Tierschutzgesetz). Animals were anesthetized by immersion in water containing 0.2% w/v tricaine (MS-222, Sigma, Deisenhofen, Germany) for 5 min, and subsequently placed on ice during surgical treatment. All efforts were made to minimize animal suffering.

### Protein expression in *Xenopus* oocytes

*Xenopus* oocytes were obtained by collagenase treatment after partial ovariectomy from *Xenopus laevis* females. cRNAs were prepared using the SP6 mMessage mMachine Kit (Agilent Technologies, Santa Clara, CA). A 50 nl aliquot containing 20–25 ng rat gastric H,K-ATPase α-subunit cRNA and 5 ng wild-type H,K-ATPase β-subunit cRNA was injected into each cell. The variant HKαS806C, which carries the mutation S806C within the M5/M6 loop to enable site-specific labeling with TMRM (see Figure S2), was used as parent construct for mutagenesis and is termed “wild-type” herein. The S806C mutation does not affect ion transport activity [32], [33]. Mutagenesis was performed by recombinant PCR and verified by DNA sequencing (Eurofins MWG Operon, Ebersberg, Germany). After cRNA injection, oocytes were kept in ORI buffer (110 mM NaCl, 5 mM KCl, 2 mM CaCl_2_, 5 mM HEPES, pH 7.4, plus 50 mg/l gentamycin) at 18°C for two days.

### Experimental solutions

Experimental solutions were Na buffer, TMA buffer, NMDG buffer, (20 mM TEACl, 5 mM BaCl_2_, 5 mM NiCl_2_ and 90 mM NaCl, TMA-Cl or NMDG-Cl, respectively). For measurements at extracellular pH (pH_ex_) of 7.4, the solution was buffered with 10 mM HEPES, whereas 10 mM MES was used for experiments at pH_ex_ 5.5. The pH of the buffers used is indicated by a lower index. For measurements in presence of extracellular K^+^, 5 mM NaCl of the Na_7.4_ buffer (or Na_5.5_ buffer) were replaced by 5 mM K^+^. For intracellular acidification measurements in Na_7.4_ buffer, 40 mM NaCl were replaced by 40 mM Na-butyrate. At pH_ex_ 7.4, a concentration of 40 mM butyrate corresponds to 100 µM undissociated (thus membrane-permeable) butyric acid, which was reported to increase cytosolic [H^+^] of oocytes from 50 nM to 160 nM (corresponding to an intracellular pH change from ∼7.3 to ∼6.8 [35]). All solutions contained 100 µM ouabain to inhibit the endogenous *Xenopus* Na,K-ATPase.

### Voltage clamp fluorometry

Site-specific labeling of H,K-ATPase α-subunit mutant S806C (HKαS806C) upon expression oocytes was achieved by incubating oocytes in Na_7.4_ buffer with 5 µM TMRM (tetramethylrhodamine-6-maleimide, Molecular Probes) for 5 min at room temperature in the dark, followed by extensive washes in dye-free Na_7.4_ buffer. Labeled oocytes were transferred into an oocyte perfusion chamber (model RC-10, Warner Instr., Hamden, CT), which was mounted on the stage of an epifluorescence microscope (Axioskop 2FS; Carl Zeiss, Göttingen, Germany) equipped with a 40× water immersion objective (numerical aperture = 0.8). Fluorescence was excited with a 100 W tungsten lamp using a 535DF50 excitation filter, a 565 EFLP emission filter and a 570DRLP dichroic mirror (Omega Optical, Battleborough, USA). Fluorescence monitoring used a PIN-022A photodiode (United Detector Technologies, Torrence, CA) mounted to the microscope camera port, whose photocurrents were amplified by a DLPCA-200 low-noise current amplifier (FEMTO Messtechnik GmbH, Berlin, Germany). Control of transmembrane voltage was achieved by means of a Turbotec 05 two-electrode voltage clamp amplifier (npi, Tamm, Germany). Fluorescence and current signals were recorded simultaneously using a Digidata 1322A interface and pClamp 9.2 software (Molecular Devices, Sunnyvale, CA).

### Rb^+^ uptake assay

Two days after injection, non-injected control oocytes and H,K-ATPase-expressing oocytes were preincubated for 15 min in TMA_7.4_ buffer, containing 100 µM ouabain for complete inhibition of the endogenous Na,K-ATPase. Oocytes were then incubated for 15 min in Rb^+^-flux-buffer (5 mM RbCl, 85 mM TMACl (NMGCl or NaCl), 20 mM TEACl, 5 mM BaCl_2_, 5 mM NiCl_2_, 10 mM MES, pH 5.5 or pH 7.4, 100 µM ouabain). For intracellular acidification measurements in Na_7.4_ buffer, 40 mM NaCl in the Rb^+^-flux-buffer were replaced by 40 mM Na-butyrate. In NMDG- or TEA-based solutions, 40 mM NMDG (or TEA) were substituted by 40 mM butyric acid prior to pH adjustment using HCl. Temperature-dependent Rb^+^ uptake measurements (between 18°C and 34°C) were performed by incubation in an HLC thermomixer (Ditabis, Pforzheim, Germany). Rb^+^ uptake under voltage control was measured using the aforementioned two-electrode voltage clamp setup to apply −100 mV membrane potential during incubation in Rb^+^-flux-buffer.

After three washing steps in Rb^+^-free TMA_7.4_ buffer and one wash in Millipore water, each individual oocyte was homogenized in 1 ml of Millipore water. 20 µl samples of the oocyte homogenates were automatically transferred into the transversely heated graphite furnace of an AAnalyst800™ atomic absorption spectrometer (Perkin Elmer, Waltham, MA). After two drying steps at 110°C and 130°C and a pyrolysis step at 500–600°C, atomization was carried out at 1700–1800°C. After each measurement, the graphite furnace was heated to 2400°C for cleaning. Rubidium absorption was measured at 780 nm using a Rubidium hollow cathode lamp (Photron, Melbourne, Australia). After Zeeman-background correction, Rb^+^ contents were calculated from the integrated peak area of the signal according to a standard calibration curve. Between 0 and 70 µg/L RbCl an excellent linearity (*r* ^2^≥0.99) was observed. The detection limit of Rb^+^ was in the upper picomolar range (characteristic mass: 10 pg).

## Results

### Effect of extra- and intracellular pH on presteady-state fluorescence changes

**Figure 2:**
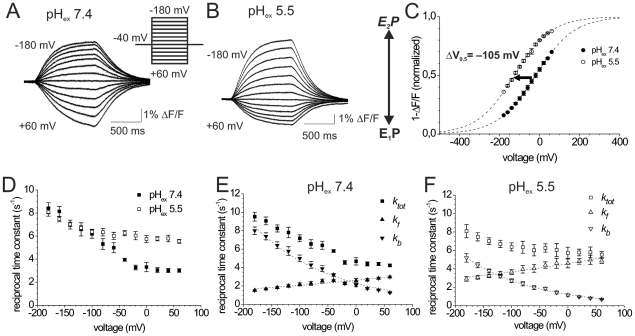
Effects of extracellular pH on the E_1_P/E_2_P distribution of gastric H,K-ATPase. (A,B) Fluorescence responses of site-specifically labeled gastric H,K-ATPase under K^+^-free conditions (90 mM Na^+^ in the extracellular solution) upon voltage jumps from −40 mV to voltages between −180 mV and +60 mV in (−20 mV steps, see inset in A) at an extracellular pH of 7.4 (A) or 5.5 (B). (C) Voltage dependence of normalized fluorescence amplitudes *(1-ΔF/F)* from experiments as in (A,B) for pH_ex_ 5.5 (○), and for pH_ex_ 7.4 (•). Data are means±S.E. of 11–14 oocytes. Superimposed as dashed lines are curves resulting from fits of a Boltzmann-type function to the data sets (pH_ex_ 7.4: *V_0.5_* = −19.7±5.4 mV, *z_q_* = 0.26±0.02; pH_ex_ 5.5: *V_0.5_* = −126.4±16.6 mV, *z_q_* = 0.27±0.04). The fluorescence amplitudes *1-ΔF/F* were normalized to the difference between the saturation values at positive or negative potentials, respectively, as obtained from the fits. (D) Reciprocal time constants (*τ^−1^*) from fits of a single exponential function to voltage jump-induced fluorescence changes under K^+^-free conditions at pH_ex_ 5.5 (□) and pH_ex_ 7.4 (▪). Data are means ± S.E. from 15–17 oocytes. (E,F) Graphs showing the forward (*k_f_*) and reverse (*k_b_*) rate constants of the E_1_P↔E_2_P transition at pH_ex_ 7.4 (E) and pH_ex_ 5.5 (F), as calculated from the observed *k_tot_ = τ^−1^* in (D) and the voltage-dependent fluorescence amplitudes in (C) according to Supporting Information (**Appendix S1**). Superimposed in (E,F) are fits of a single exponential function to the calculated *k_f_* and *k_b_* values. The resulting fit parameters (rate constants at 0 mV: *k_b_*(0), *k_f_*(0), and *z_q_* values), as summarized in Table 1, were: pH_ex_ 7.4 (E): *k_f_(0)* = 2.61±0.05 s^−1^, *z_q,f_* = −0.06±0.01; *k_b_(0)* = 2.31±0.10 s^−1^, *z_q,b_* = 0.21±0.01; pH_ex_ 5.5 (F): *k_f_(0)* = 4.56±0.05 s^−1^, *z_q,f_* = −0.056±0.003; *k_b_(0)* = 1.22±0.10 s^−1^, *z_q,b_* = 0.230±0.004.

The aim of this study was to investigate electrogenic partial reactions within the H^+^ translocating branch of gastric H,K-ATPase and to scrutinize, whether and how the concept of high-field access channels established for Na,K-ATPase [12] can be transferred to the H^+^ pump. For that purpose, we performed voltage clamp fluorometry on gastric H,K-ATPase mutant S806C under various ionic conditions. Figure 2A shows typical fluorescence signals at pH_ex_ 7.4 resulting from voltage pulses to a series of test potentials between −180 and +60 mV, which were recorded in the absence of K^+^ and with 90 mM Na^+^ in the extracellular solution. The fluorescence of the dye attached to the extracellular end of helix M5 (see structural model in Figure S2), increases upon jumps to negative potentials and decreases at depolarizing membrane voltage. In analogy to Na,K-ATPase, positive voltages should favor the transition to E_2_P, whereas negative voltage steps drive the enzyme into E_1_P. According to the crystal structures of several reaction intermediates of the related SERCA Ca^2+^-ATPase [36], [37], [38], [39], the central helix M5 moves in relation to the surrounding helices during the cycle. Since TMRM is sensitive to hydrophobicity and collisional quenching, the observed fluorescence signals presumably result from a motion of the extracellular end of M5 from a buried, sheltered environment in E_1_P into a more aqueous, quenching environment in E_2_P.

Although it would be desirable to study the H^+^ pump under physiological working conditions (i.e. pH_ex_ down to ∼1), it was not possible to apply such large [H^+^] gradients in our experiments. The recording of a single set of VCF traces as shown in Fig. 2A requires absolutely stable fluorescence for at least 90 s, but at pH values lower than 5.5 the signal quality was too poor for kinetic analyses. Furthermore, as we show below, extracellular acidification leads to significant proton leakage into the cells, which not only impairs long-term stability of the cells, but also leads to an ill-defined [H^+^] gradient. For the sake of reliable working conditions, we had to restrict our analyses to the pH_ex_ range between 7.4 and 5.5, which - in terms of H^+^ concentration - is still an about 100-fold difference.

A change in the pH_ex_ from 7.4 to 5.5 altered the fluorescence signals profoundly. At pH_ex_ 7.4 (Fig. 2A), the largest fluorescence changes occurred at positive voltages, at which the transition to E_2_P should be favored, whereas the opposite was observed at pH_ex_ 5.5 (Fig. 2B), with fluorescence changes being largest upon negative voltage steps, which should drive the enzyme into E_1_P. Plotting the steady-state fluorescence amplitudes against the membrane potential resulted in sigmoidal *(1-ΔF/F)-V* distributions, which could be approximated by a Boltzmann-type function (Fig. 2C). Of note, the pH_ex_ change from 7.4 to 5.5 resulted in a strong negative *V_0.5_* shift of the *(1-ΔF/F)-V* distribution by −105 mV (Fig. 2C and Table 1). This observation is puzzling since it would indicate that an increase of the extracellular proton concentration increases formation of E_2_P, in contrast to established paradigms about the electrogenicity of Na,K-ATPase. For the Na^+^ pump, an increase of [Na^+^]_ex_ shifts the voltage-dependent distribution of the slow charge from ouabain-sensitive transient currents (Rakowski, 1993, Holmgren et al. 2000) towards positive potentials. Hence, high [Na^+^]_ex_ drives the E_1_P/E_2_P equilibrium of the Na^+^ pump towards E_1_P, in line with the concept of an extracellular access channel for Na^+^ ions.

**Table 1:** Parameters characterizing the voltage dependence of the E1P↔E2P conformational transition.

cation, pH	*V_0.5_*/mV	*z_q_*	*k_f_(0)*/s^−1^	*z_q,f_*	*k_b_(0)*/s^−1^	*z_q,b_*
Na^+^, pH_ex_7.4	−19.7±5.4	0.26±0.02	2.61±0.05	0.06±0.01	2.31±0.10	0.21±0.01
TMA^+^, pH_ex_7.4	−89.6±3.3	0.48±0.04	3.34±0.12	0.11±0.01	0.87±0.07	0.30±0.02
Na^+^, pH_ex_7.4	−126.4±16.6	0.27±0.03	4.56±0.06	0.06±0.01	1.22±0.02	0.23±0.01
TMA^+^, pH_ex_7.4	−125.2±11.4	0.49±0.07	3.98±0.18	0.09±0.02	0.53±0.06	0.33±0.02

Parameters from fits of a Boltzmann-type function to the data in Fig. 2C and 5A,B (*V_0.5_* and *z_q_*) and parameters characterizing the voltage dependence of forward and backward rate constants *k_f_* and *k_b_* from data in Figure 2E,F and 5D,E,G,H (see **Appendix S1**).

### pH effects on presteady-state kinetics of gastric H,K-ATPase

To delineate the processes underlying this behavior of the H,K-ATPase, we analyzed the kinetics of the voltage step-induced conformational changes. Figure 2D shows the voltage dependence of the reciprocal time constants (*τ^−1^*) obtained from fitting single exponential functions to the fluorescence signals at the two different pH_ex_ values. Assuming that the fluorescence signals directly reflect the redistribution between E_1_P and E_2_P in response to voltage steps, a simplified two-state kinetic model can be used to derive information about the kinetics of the forward and the backward reaction (see **Appendix S1** and [40]). Within this framework, the observed reciprocal time constants (*τ^−1^ = k_tot_*) represent the sum of the voltage-dependent rate constants for the forward (*k_f_*) and the backward reaction (*k_b_*) of the E_1_P↔E_2_P transition (*τ^−1^ = k_tot_ = k_f_+k_b_*), which is coupled to extracellular cation uptake or release steps. From the actual poise of the E_1_P/E_2_P distribution (reflected by the *(1-ΔF/F)-V* curve) and *k_tot_*, the individual forward and backward rate constants *k_f_* and *k_b_* at each membrane potential *V* can be calculated (Eq. A6 and Eq. A7 in **Appendix S1**). Subsequently, the voltage-dependent values *k_f_(V)* and *k_b_(V)* can be fitted by a single exponential function (according to Eq. A1 and Eq. A2 in **Appendix S1**). From these fits, the parameters characterizing the voltage dependence of *k_f_* and *k_b_* can be determined, such as the values for the equivalent charge, *z_q,f_* and *z_q,b_*, and the rate constants at 0 mV membrane voltage, *k_f_(0)* and *k_b_(0)*. Table 1 summarizes these parameters for all data sets analyzed in this work. Notably, at strongly negative potentials, the reciprocal time constants (*τ^−1^ = k_tot_*) were similar for both pH_ex_ values (Fig. 2D). Since the total rate constant *k_tot_* at negative potentials should mainly be determined by *k_b_* of the backward reaction, this observation indicates that extracellular acidification does not accelerate the reverse reaction (E_2_P→E_1_P). The calculated *k_b_* values (Fig. 2E and 2F) are even lower at acidic pH_ex_, with *k_b_(0)* values showing a reduction by about 50% upon a change from pH_ex_ 7.4 to 5.5 (Table 1). At positive voltages, however, the reciprocal time constants (*τ^−1^ = k_tot_*) at pH_ex_ 5.5 were nearly two-fold larger than at pH_ex_ 7.4 (Fig. 2D), which is reflected by a similar increase in *k_f_* (Fig. 2E,F and Table 1 for *k_b_(0)* and *k_f_(0)* values). These observations indicate an acceleration of the *forward* reaction (E_1_P→E_2_P) by extracellular *acidification*, again contradicting the expectations from an extracellular access channel concept. The slope factors *z_q,b_*, which characterize the voltage dependence of the *k_b_* values in Fig. 2E and 2F, are very similar. Notably, the backward rate constant carries most of the voltage dependence (*z_q,b_* values 0.21 to 0.23, similar to the slope factor *z_q_* from fits of the corresponding *(1-ΔF/F)-V* curves with a Boltzmann-type function), whereas the forward reaction is only weakly voltage-dependent (*z_q,f_* values 0.05 to 0.06).

### Effects of intracellular pH changes on the E_1_P/E_2_P distribution

**Figure 3:**
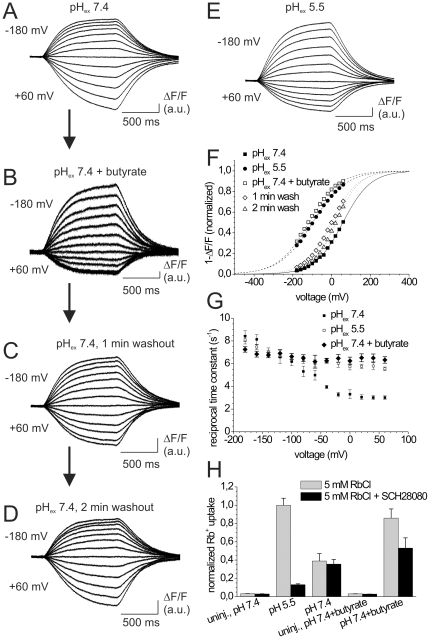
Effects of intracellular acidification on the E_1_P/E_2_P distribution and Rb^+^ transport. (A–E) Fluorescence responses of TMRM-labeled HKαS806C/βWT under extracellular K^+^-free conditions (90 mM extracellular Na^+^) upon voltage jumps from −40 mV to potentials between −180 mV and +60 mV in −20 mV steps. Recordings in (A–D) originated from a single oocyte (A) at pH_ex_ 7.4, (B) after 1 min in presence of 40 mM Na-butyrate (pH_ex_ 7.4), and after 1 min (C) and 2 min (D) washout of butyrate (pH_ex_ 7.4 buffer). (E) Fluorescence responses from a different cell in pH_ex_ 5.5 buffer. (F) Voltage dependence of stationary fluorescence amplitudes *1-ΔF/F* from the recordings in (A–E) at pH_ex_ 7.4 (▪), pH_ex_ 7.4+40 mM butyrate (□), and after 1 min (⋄) or 2 min (▵) washout of butyrate. Data at pH_ex_ 5.5 (•) are also shown. Fits of a Boltzmann-type function are superimposed to each data set, and the fluorescence amplitudes were normalized to saturation values from the fits. (G) Reciprocal time constants (*τ^−1^*) from fits of a single exponential function to fluorescence changes under K^+^-free conditions. Data obtained at pH_ex_ 7.4 in the presence of 40 mM butyrate (♦) are compared to those in butyrate-free solutions at pH_ex_ 5.5 (□) and at pH_ex_ 7.4 (▪). Data are means±S.E. from 12–17 oocytes. (H) H,K-ATPase-mediated Rb^+^ uptake (at 5 mM Rb^+^) measured on individual cells by atomic absorption spectroscopy in the absence (gray) or presence (black) of 10 µM SCH28080 at different pH_ex_ and ionic conditions. Results from non-injected and HKαS806C/βWT-expressing oocytes at pH_ex_ 5.5, pH_ex_ 7.4, and pH_ex_ 7.4+40 mM butyrate are shown. Data are means±S.E. from three experiments on different cell batches with 15–20 oocytes per condition, and normalized to the Rb^+^ uptake of HKαS806C/βWT at pH_ex_ 5.5 (mean specific activities of 15.4, 23.1 and 39.0 pmol/oocyte/min).

Since the H,K-ATPase binds protons intracellularly at around pH 7 but releases them extracellularly against a luminal pH of up to ∼1, it is conceivable that the proton pump might be rather unaffected by pH_ex_ changes from 7.4 to 5.5, whereas the enzyme may be much more sensitive to minute pH changes at the intracellular side. Therefore, we asked whether the observed *V_0.5_* shifts of the *(1-ΔF/F)-V* curves could be due to an influence of the extracellular pH on the pH inside the oocytes. In fact, several studies on *Xenopus* oocytes reported small pH_in_ changes upon extracellular acidification [35], [41], [42], with pH_ex_ 5.5 causing a drop in the intracellular pH by about 0.5 units [42]. To test this hypothesis experimentally, we carried out an “acid-bath procedure” by adding the weak organic acid butyric acid to the extracellular solution. Butyric acid can permeate the plasma membrane in its neutral form and dissociate intracellularly, thereby allowing a controlled intracellular acidification to be achieved (see Material and Methods). To acidify the oocyte interior by ∼0.5 pH units, 40 mM NaCl was replaced by an equal amount of Na-butyrate at pH_ex_ 7.4, as pioneered elsewhere [35]. In Figure 3, the effects on the voltage-dependent fluorescence signals after a solution exchange from butyrate-free (Fig. 3A) to a butyrate-containing solution (Fig. 3B), and back to butyrate-free solution (Fig. 3C,D) are shown. Notably, the fluorescence changes at pH_ex_ 7.4 in presence of 40 mM butyrate (Fig. 3B) were very similar to the ones observed at pH_ex_ 5.5 (Fig. 3E). The *(1-ΔF/F)-V* curves (Fig. 3F) show that at the same pH_ex_ of 7.4, the *V_0.5_* value of the conformational distribution in presence of butyrate is shifted to negative potentials in essentially the same way as observed at pH_ex_ 5.5 in butyrate-free solution. This supports the hypothesis that the observed *V_0.5_* shift of the *(1-ΔF/F)-V* distribution at pH_ex_ 5.5 entirely results from intracellular acidification. The concept that the H,K-ATPase is tightly regulated by intracellular pH is further supported by the fact that the reciprocal rate constants of the E_1_P↔E_2_P relaxation in the presence of 40 mM butyrate at pH_ex_ 7.4 were very similar to those measured at pH_ex_ 5.5 (Fig. 3G). As already outlined for the rate constants at pH_ex_ 5.5 (Fig. 2F), the observed increase in *k_tot_* at positive potentials must largely be due to an increased rate constant *k_f_* for the forward reaction, for which a dependence on intracellular pH is rather straightforward. The effects of butyrate on intracellular pH and the conformational distribution of the gastric H,K-ATPase were strictly reversible. Already a few minutes after a solution exchange to butyrate-free solution (Fig. 3C,D), the fluorescence signals were almost identical to the initially observed fluorescence signals at pH_ex_ 7.4 (Fig. 3A), in agreement with a time constant of ∼315 s determined by Stewart *et al.* for the recovery of intracellular pH after withdrawal of butyrate [35].

### Effect of extra- and intracellular pH on steady-state cation pumping

To test the significance of the described pH effects on stationary H^+^/K^+^ exchange transport, we measured the Rb^+^ transport activity of the H,K-ATPase under turnover conditions at saturating Rb^+^ concentrations (5 mM). As shown in Figure 3H, Rb^+^ uptake of the gastric H,K-ATPase at pH_ex_ 5.5 was more than two-fold larger than the transport activity at pH_ex_ 7.4. Again, the effect of the extracellular acidification could be attributed to an intracellular pH decrease, since almost the same increase in transport activity was observed at pH_ex_ 7.4 in presence of 40 mM butyrate. These findings strongly suggests that the availability of protons at the intracellular side is not only rate-limiting for the E_1_P→E_2_P transition (Fig. 3G), but also for the turnover rate during stationary cation pumping. Notably, the relatively increased E_1_P preference of the enzyme at neutral intracellular pH (pH_ex_ 7.4 without butyrate, Fig. 2C) compared to conditions of slight intracellular acidification (pH_ex_ 5.5 or pH_ex_ 7.4 with butyrate) was also reflected by distinct differences regarding the inhibition by 10 µM SCH28080 (black bars in Fig. 3H). To understand the effects of this reversible, E_2_/E_2_P-specific, K^+^-competitive inhibitor, two aspects have to be kept in mind. First it must be considered that only the protonated form of SCH28080 is pharmacologically active, with deprotonation occuring with a pK_a_ of 5.5 [43], [44]. Consequently, at pH_ex_ 7.4 only about 1% of the total inhibitor concentration (i.e. ∼0.1 µM) is in the protonated, active form, which is in the same range as the IC_50_ values determined for this K^+^-competitive antagonist (67 nM for [K^+^] = 0, and 480 nM for [K^+^] = 10 mM [45]; 0.18 µM and 0.66 µM for K^+^-stimulated ATPase activity [43]), whereas at pH_ex_ 5.5 about 50% of the compound is active. The second important point is the fractional amount of enzyme molecules in E_2_ or E_2_P, because the inhibitor is specific for these intermediates. Therefore, the highly effective inhibition at pH_ex_ 5.5 is on one hand due to the higher abundance of the active compound, and on the other hand due to the strong shift towards the E_2_P-state (at potentials around −10 mV, which are relevant for the Rb^+^ flux measurements without voltage control, see Fig. 2C and 3F) caused by the concomitant decrease in intracellular pH. However, the higher extent of SCH28080 inhibition at the same pH_ex_ of 7.4, depending on whether the cytoplasm is acidified by the addition of butyrate or not (Fig. 3H, black bars), indicates that intracellular acidification indeed entails a higher E_2_P preference of the H,K-ATPase.

### Changes in the conformational distribution in response to extracellular K^+^

**Figure 4:**
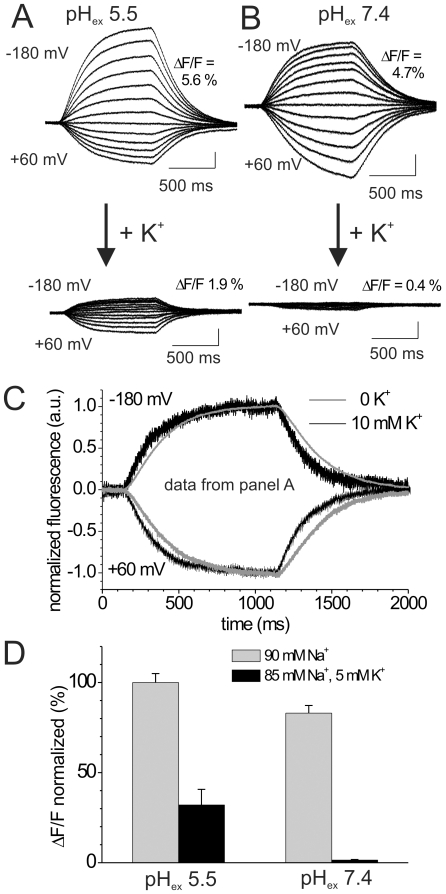
Effects of extracellular K^+^ on voltage-dependent fluorescence changes. (A,B) Voltage step-induced fluorescence responses of TMRM-labeled oocytes expressing HKαS806C/βWT in K^+^-free (upper traces) or 5 mM K^+^-containing extracellular solution (lower traces) at pH_ex_ 5.5 (A), and pH_ex_ 7.4 (B), according to a voltage protocol as in Fig. 2A (inset). (C) Comparison of the time course of the fluorescence signals from panel (A) in response to voltage pulses to −180 mV and +60 mV in the absence of K^+^ and in the presence of 5 mM K^+^. Signals were normalized to the fluorescence amplitude reached at the end of the voltage pulse to −180 mV or +60 mV, respectively. (D) Comparison of normalized ΔF/F values (change in stationary fluorescence between −180 mV and +60 mV, divided by fluorescence at −40 mV) in the absence (gray bars) or presence (black bars) of 5 mM extracellular K^+^, both at pH_ex_ 5.5 and pH_ex_ 7.4. Data are means±S.D. from 3–5 oocytes, normalized to the mean ΔF/F at pH_ex_ 5.5 in K^+^-free solution.

Of note, all VCF experiments shown so far were done under K^+^/Rb^+^-free conditions and therefore do not reflect the conditions of the Rb^+^ fluxes in Fig. 3H. In order to discriminate the effects of different monovalent cations on the E_1_P/E_2_P distribution, we first measured the changes of the voltage-dependent fluorescence signals under H^+^/K^+^ turnover conditions (Fig. 4). After a change from K^+^-free solution to 5 mM K^+^, the magnitudes of fluorescence changes were substantially reduced at both investigated pH_ex_ values (Fig. 4A,B), with the effect being more pronounced at pH_ex_ 7.4 (compare black bars in Fig. 4D). This is very similar to the effect of K^+^ on the fluorescence changes of TMRM-labeled Na,K-ATPase [31]. Cyclic turnover at high K^+^ concentrations results in a redistribution of enzyme molecules over all reaction cycle intermediates, thereby increasing the accumulation of states (e.g. dephosphorylated E_1_-type intermediates), whose occupancies are insensitive to transmembrane voltage. This, in effect, diminishes the number of pump molecules that contribute to the fluorescence changes related to the voltage-dependent redistribution between E_1_P and E_2_P states. Due to the proposed acceleration of the rate-limiting step early within the H^+^ branch of the cycle by slight intracellular acidification (as a result of the pH_ex_ change to 5.5), fewer molecules are kinetically trapped in voltage-insensitive intermediates, which explains the significantly larger fluorescence changes under turnover conditions at pH_ex_ 5.5. A comparison of the time course of the fluorescence responses to −180 mV and +60 mV from Figure 4A in the absence of K^+^ and at 5 mM K^+^ (see normalized signals in Fig. 4C) shows that K^+^ accelerates the conformational relaxation at negative as well as positive potentials. This global acceleration of rate constants indicates that K^+^ opens up a second relaxation pathway (E_2_P→E_2_→E_1_) that occurs in addition to the E_1_P↔E_2_P relaxation.

### Na^+^ effects on presteady-state fluorescence changes of gastric H,K-ATPase

**Figure 5:**
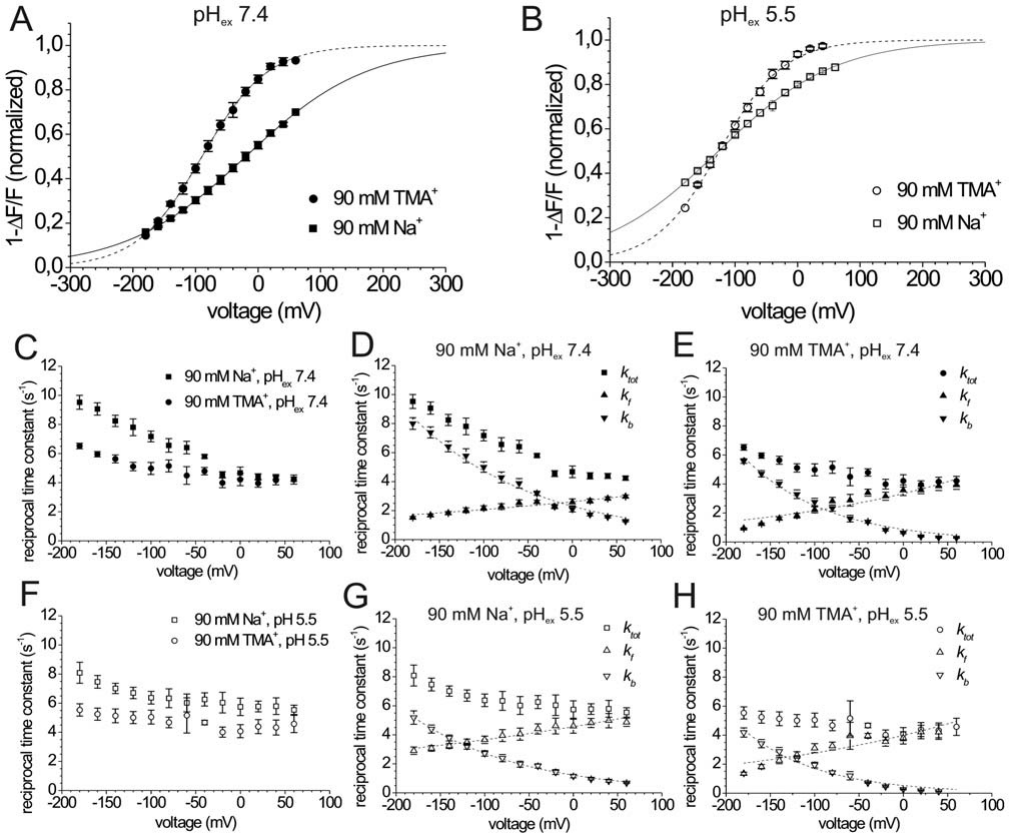
Effect of extracellular Na^+^ on the E_1_P↔E_2_P conformational transition. (A,B) Voltage dependence of fluorescence amplitudes *1-ΔF/F* of TMRM-labeled HKαS806C/βWT at pH_ex_ 7.4 (A), and pH_ex_ 5.5 (B) in the presence of 90 mM extracellular Na^+^ (▪,□) compared to Na^+^-free conditions (•,○; Na^+^ replacement by 90 mM TMA^+^). Data are means±S.E. of 13–15 oocytes. Superimposed are curves resulting from a fits of a Boltzmann-type function to the data (pH_ex_ 7.4, 90 mM TMA^+^: *V_0.5_* = −89.6±3.3 mV, *z_q_* = 0.48±0.04; pH_ex_ 7.4, 90 mM Na^+^: *V_0.5_* = −19.7±5.4 mV, *z_q_* = 0.26±0.02; pH_ex_ 5.5, 90 mM TMA^+^: *V_0.5_* = −125.2±11.4 mV, *z_q_* = 0.49±0.07; pH_ex_ 5.5, 90 mM Na^+^: *V_0.5_* = −126.4±16.6 mV, *z_q_* = 0.26±0.03), parameters are listed in Table 1. The fluorescence amplitudes were normalized to the saturation values from the fits. (C,F) Reciprocal time constants (*τ^−1^*) from fits of a single exponential function to fluorescence signals in Na^+^-free and in 90 mM Na^+^-containing solutions for pH_ex_ 7.4 (C) and pH_ex_ 5.5 (F). Data are means±S.E. from 13–15 oocytes. (D,E) Calculated forward (*k_f_*) and reverse (*k_b_*) rate constants of the E_1_P↔E_2_P transition in the presence of 90 mM Na^+^ (D), and in Na^+^-free solution (E) at pH_ex_ 7.4, as calculated from the observed *k_tot_ = τ^−1^* values in (C) and the voltage-dependent fluorescence amplitudes in (A) according to Supporting Information (**Appendix S1**). (G,H) Calculated forward (*k_f_*) and reverse (*k_b_*) rate constants at pH_ex_ 5.5 in the presence of 90 mM Na^+^ (G) and in Na^+^-free solution (H), as calculated from the *k_tot_ = τ^−1^* values in (F) and fluorescence amplitudes in (B). Superimposed in (D,E,G,H) are fits of a single exponential function to the *k_f_* and *k_b_* values, the resulting fit parameters (*k_b_*(0), *k_f_*(0), and *z_q_* values) are summarized in Table 1.

In a previous publication, we found indications for a competition between Na^+^ and Rb^+^ at the extracellular binding sites [28], since the apparent affinity for extracellular Rb^+^ in Rb^+^ uptake experiments was reduced about 7-fold in the presence of extracellular Na^+^. To scrutinize, whether such competitive effects of extracellular Na^+^ ions also affect the voltage dependence and kinetics of the E_1_P↔E_2_P transition, we compared the voltage dependence of the fluorescence amplitudes and of the respective reciprocal time constants in extracellular Na^+^-free and Na^+^-containing solutions (Fig. 5). Notably, the parameters *z_q_* for the *(1-ΔF/F)*-V distributions were larger in the absence than in the presence of Na^+^ at both pH_ex_ values (Fig. 5A,B), which will be rationalized in the Discussion and Supporting Information (**Appendix S2** and **Appendix S3**). Furthermore, the presence of extracellular Na^+^ had a large effect on the *V_0.5_* values of the E_1_P/E_2_P distribution at pH_ex_ 7.4 (Fig. 5A and Table 1), but not at pH_ex_ 5.5 (Fig. 5B and Table 1). From the voltage dependence of the reciprocal time constants (*τ^−1^* = *k_tot_*) (Fig. 5C,F) and the *k_f_* and *k_b_* values calculated thereof (Fig. 5D,E and Fig. 5G,H), it is evident that extracellular Na^+^ accelerates the reverse rate constants *k_b_(0)* by a factor of ∼2.5 at both pH_ex_ values. This indicates that extracellular Na^+^ ions, which are by far more abundant than protons at pH_ex_ 7.4 as well as 5.5, can act as H^+^ analogs when the binding sites face the extracellular medium (see Discussion). In contrast, *k_f_(0)* is only slightly changed (∼22% decrease at pH_ex_ 7.4, Fig. 5D,E; ∼15% increase at pH_ex_ 5.5, Fig. 5G,H) compared to Na^+^-free conditions (Table 1). Thus, extracellular Na^+^ mainly accelerates the reverse rate constant *k_b_*, whereas *k_f_* is essentially unchanged, and the total rate constant is consequently increased only at negative voltages, in notable contrast to the effect of K^+^ on the relaxation kinetics (see Discussion).

### Temperature dependence of steady-state pump activity

**Figure 6:**
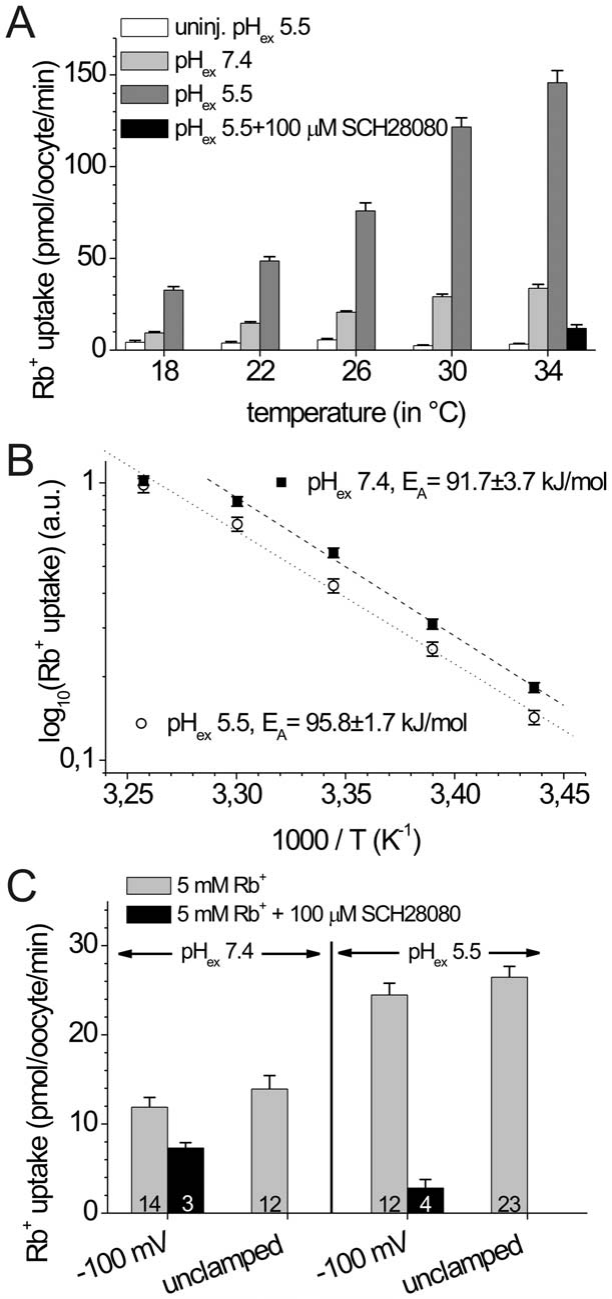
Temperature and voltage dependence of Rb^+^ uptake by gastric H,K-ATPase. (A) H,K-ATPase-mediated Rb^+^ uptake (in pmol/oocyte/min) at 5 mM Rb^+^ and a pH_ex_ of 7.4 (light gray bars) or 5.5 (gray bars) at temperatures between 18 and 34°C, as indicated. White bars represent Rb^+^ uptake of non-injected control oocytes at each temperature and pH_ex_ 5.5. The black bar at 34°C shows the residual Rb^+^ uptake at pH_ex_ 5.5 in the presence of 100 µM SCH28080. Data in each column are means of 20–25 oocytes from oocytes of one cell batch. (B) Arrhenius plot for temperature-dependent Rb^+^ uptakes from data as in (A) at pH_ex_ 7.4 (▪), and pH_ex_ 5.5 (○). Data represent means±S.E. of three independent experiments (similar to the one shown in A), after normalization to Rb^+^ uptake at 34°C for each experiment. Activation energies obtained from linear fits to the data (superimposed lines) are given for each pH_ex_. (C) Rb^+^ uptake (in pmol/oocyte/min) at 5 mM Rb^+^ and pH_ex_ 7.4 or 5.5 for oocytes expressing HKαS806C/βWT, which had either been clamped to a membrane potential of −100 mV, or subjected to Rb^+^ uptake without voltage clamping (V_m_∼−10 to −20 mV). Black bars represent Rb^+^ uptake of H,K-ATPase-expressing oocytes clamped at −100 mV in the presence of 100 µM SCH28080. Data are means±S.D. from several oocytes of a single batch (numbers stated on each column).

Rb^+^ uptake measurements were carried out at different temperatures to determine the activation energy of Rb^+^ transport at pH_ex_ 5.5 and pH_ex_ 7.4. As shown in Figure 6A, the Rb^+^ transport activity at pH_ex_ 5.5 was substantially larger than at pH_ex_ 7.4 in the whole temperature range covered by our experiments (18–34°C). Arrhenius plots yielded linear relationships at both investigated pH (Fig. 6B). At pH_ex_ 7.4, we consistently observed in several independent experiments that the data points corresponding to a temperature of 34°C significantly diverged from the linear function defined by the other data points. Such a behavior is not uncommon, as exemplified by the temperature dependence of K^+^-stimulated pump currents of Na,K-ATPase expressed in oocytes, for which a reduced slope of the Arrhenius plot at temperatures above 26°C was observed too [46]. Exclusion of the data point for 34°C at pH_ex_ 7.4 yielded activation energies of similar magnitude at both pH_ex_ conditions (95.8±1.7 kJ at pH_ex_ 5.5 versus 91.7±3.7 kJ at pH_ex_ 7.4). The close similarity of these values suggests that Rb^+^ uptake of the gastric proton pump at both pH values is rate-limited by the same partial reaction, and due to the high activation energy, this step is likely not to be diffusion-controlled, but might be related to a major conformational change.

### Voltage dependence of steady-state cation transport

To assess the voltage dependence of the overall pump activity, we performed Rb^+^ uptake experiments also under transmembrane voltage control (Na^+^-free conditions). In Figure 6C, the Rb^+^ uptake activity at saturating Rb^+^ concentrations (5 mM) in not voltage-clamped oocytes (V_m_∼−10 to −20 mV, determined in independent experiments) is compared to the Rb^+^ uptake activity of oocytes whose membrane potential was clamped to −100 mV by two-electrode voltage clamping. At pH_ex_ 7.4 as well as pH_ex_ 5.5, only a slight and hardly significant decrease of the Rb^+^ transport activity was observed at −100 mV compared to unclamped oocytes. Importantly, however, the about two-fold increase of Rb^+^ transport at pH_ex_ 5.5 compared to pH_ex_ 7.4, as observed previously in Fig. 3H, occurred irrespective of the membrane potential. This finding supports the hypothesis that an intracellular pH-sensitive and only weakly voltage-dependent event is not only rate-limiting for the E_1_P→E_2_P conformational transition (monitored by the VCF experiments) but also for the overall pumping rate.

## Discussion

### Effects of extracellular pH on the E_1_P/E_2_P distribution

It is generally accepted that the transport cycle of the H,K-ATPase proceeds according to a Post-Albers-type reaction scheme, as formulated for the Na,K-ATPase, despite some difference in detail, such as the strong E_2_P preference of the gastric proton pump under physiological conditions [26], [28], [47], [48]. Although the H,K-ATPase carries out net electroneutral transport, experiments using H,K-ATPase-containing parietal cell membrane fragments attached to black lipid membranes have shown transient current signals upon ATP concentrations jumps in the absence of K^+^ [19], [20], [21] suggesting that an electrogenic event takes place during H^+^ translocation. Thus, as a first approach, one could assume that electrogenicity in the H,K- and the Na,K-ATPase follows the same mechanism. For the Na^+^ pump, the slowest phase of presteady-state Na^+^ movement, which is kinetically coupled to the E_1_P↔E_2_P transition, arises from extracellular Na^+^ release from (or reverse binding to) a site located at ∼70% of the electrical distance from the extracellular side. According to the high-field access channel hypothesis, changes in membrane potential are kinetically equivalent to changes in the ‘effective’ ion concentration at the binding sites deep within the ion well [49], [50]. Thus, an about 100-fold increase in the extracellular H^+^ concentration (change from pH_ex_ 7.4 to 5.5) should shift the *V_0.5_* value of the E_1_P/E_2_P distribution towards E_1_P. The resulting shift (*ΔV_0.5_*) could then be predicted from a Nernst-like equation [9], [12], [50]:
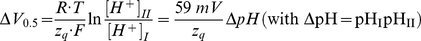
(1)Thus, using an equivalent charge or fractional well depth (*z_q_*) of 0.26–0.27 as derived from the Boltzmann curve parameters in Fig. 2C, a pH_ex_ change from 7.4 to 5.5 should result in a *positive ΔV_0.5_* of 415 mV. However, in contrast to the expectations for an extracellular H^+^ access channel, our VCF data show that a ΔpH_ex_ of 1.9 units shifts *V_0.5_* by about −105 mV.

To understand why a ΔpH_ex_ of 1.9 units does not cause a shift towards E_1_P, it must be considered that the H,K-ATPase *in situ* releases protons against a luminal pH below 1 ([H^+^]∼150 mM in the stomach), which implies that extracellular proton release from the binding pocket occurs with a pK_a_ value even lower than 1. Thus, even at a pH_ex_ of 5.5, the proton concentration is by several orders of magnitude too small to achieve a sufficient occupancy at the luminal-facing H^+^-binding site(s), which would be a prerequisite for an E_1_P shift of the E_1_P/E_2_P distribution. Even a transmembrane voltage of −200 mV would increase the effective proton concentration at the bottom of an extracellular access channel with a fractional depth *z_q_* of 0.26 (Fig. 2C) by only about 8-fold, resulting in a still insufficient ‘effective’ pH of 4.6, which is still far from the physiological pH of ∼1–2. With Na,K-ATPase, the conditions for the study of electrogenic Na^+^ transport are more favorable, since. the extracellular Na^+^ affinity of the Na^+^ pump is in the order of several hundreds of mM [7], [12], and Na^+^ concentrations in this range can easily be applied in electrophysiological experiments. Unfortunately, pH_ex_ 1 (equivalent to [H^+^] = 100 mM) cannot be tested in *Xenopus* oocyte experiments so that the question whether protons traverse an extracellular ion well cannot be resolved. However, due to the effects of extracellular Na^+^ ions on the conformational distribution the existence of an extracellular access channel of the H,K-ATPase cannot be ruled out, as discussed below.

### Intracellular pH strongly influences kinetics and poise of the E_1_P/E_2_P distribution

To explain the negative *V_0.5_* shift of the *(1-ΔF/F)-V* distribution in response to pH changes (Fig. 2C), our experiments designed to achieve a controlled intracellular acidification show that the observed *V_0.5_* shift can entirely be attributed to a slight intracellular acidification that is induced by an extracellular pH change. In fact, the *(1-ΔF/F)-V* curves (Fig. 3F) as well as the reciprocal rate constants (Fig. 3G) of the fluorescence signals at pH_ex_ 5.5 and at pH_ex_ 7.4 in the presence of 40 mM butyrate (which lowers pH_in_ by ∼0.5 units) are fully equivalent. Therefore, the extracellular pH (between 7.4 and 5.5) is apparently irrelevant for the poise of the E_1_P/E_2_P distribution, whereas already a small deviation from a neutral intracellular pH produces a large effect. The dependence on pH_in_ is in line with an intracellular access channel for protons. Indeed, the calculation of *ΔV_0.5_* (Eq. 1) according to an intracellular pH change by 0.4–0.5 units and an ‘effective’ *z_q_* of 0.26–0.27 (in the presence of 90 mM extracellular [Na^+^]) yields a *ΔV_0.5_* of about −90 to −110 mV, which is in good agreement to the observed shift of −105 mV (Fig. 2C and 3F). For the *(1-ΔF/F)-V* distributions measured in the absence of extracellular Na^+^ (Fig. 5A,B), the observed *ΔV_0.5_* (−35 mV) agrees less well with theoretical values (−48 to −61 mV, with *z_q_* between 0.48 and 0.49). However, considering the strongly negative *V_0.5_* values of the *(1-ΔF/F)-V* curves in question, it must be noted that oocyte TEVC experiments at voltages below −180 mV become increasingly problematic.

### Extracellular Na^+^ ions compete with protons for access to E_2_P, and kinetic analysis elicits the fractional depth of intra- and extracellular access channels

Since extracellular Na^+^ ions not only reduce the apparent affinity for extracellular Rb^+^ as K^+^ congeners in Rb^+^ uptake studies [28], but also profoundly change the conformational distribution (compared to the relatively small effect achieved by extracellular acidification, Fig. 5A,B), an extracellular cation access channel still has to be considered for the H,K-ATPase. At pH_ex_ 7.4, a Na^+^ concentration of 90 mM leads to a stronger accumulation of E_1_P at physiological potentials (around −70 mV), as actually expected for high [H^+^], by a combined effect on *V_0.5_* and a decrease in the slope factor *z_q_* (Fig. 5A,B). The larger fraction of E_1_P correlates with an increase of the reciprocal rate constants at hyperpolarizing potentials (Fig. 5C), which indicates an increase of the rate constant for the backward reaction (Fig. 5D,E), whereas the forward rate constant is hardly changed. These observations agree with the notion that Na^+^ ions, which are 10^4^- to 10^6^-fold more abundant than protons in our experiments, can act as H^+^ analogs within an extracellular-facing ion well. At pH_ex_ 5.5, the Na^+^ effect on *V_0.5_* of the conformational distribution was no longer present (Fig. 5B), although the reciprocal time constants at negative potentials were also increased (Fig. 5F) suggesting that the effect of Na^+^ ions on the E_1_P↔E_2_P kinetics is present even upon a 100-fold increase of the extracellular [H^+^]. But, at this lower pH_ex_ of 5.5, the E_1_P-shifting effect of the increased *k_b_* values is counteracted by the simultaneous increase of the forward rate constants (Fig. 5G,H) that occurs due to the intracellular acidification. The fact that Na^+^ ions exert H^+^-like effects on H,K-ATPase is another example for the promiscuity of the external-facing cation binding sites in P-type pumps, as outlined recently for Na,K-ATPase, in which some alkali metal ions or monovalent organic cations were shown to induce Na^+^-like or K^+^-like functional effects [51].

Since it is reported in the literature that for both Na,K- and H,K-ATPase Na^+^ ions can mimic the effect of K^+^ ions in the dephosphorylation limb of the cycle [27], one could argue that the observed kinetic effects of Na^+^ on the conformational distribution might be due to an alternative reaction branch. However, if Na^+^ ions would act like K^+^ ions to stimulate the E_2_P→E_2_→E_1_ pathway, Na^+^ addition should result in a global increase of the total relaxation rate constant (as indeed seen for K^+^, Fig. 4C), which is not observed. In fact, Na^+^ mainly affects *k_b_*, but not *k_f_*, and thus increases the total rate only at negative potentials (Fig. 5). Furthermore, a significant entry of enzyme molecules into the K^+^ limb of the cycle should lead to an accumulation of E_1_ states, which cannot contribute to the voltage-dependent E_1_P↔E_2_P relaxation. Thus, similar to the results of K^+^ addition in Fig. 4A,B, the absolute fluorescence amplitudes should decrease, which is also not observed with Na^+^. Therefore, we conclude here that under the conditions of our experiments there is no indication for a significant effect of Na^+^ on the dephosphorylation branch of the H,K-ATPase cycle.

Notably, at both investigated pH_ex_, Na^+^ had a strong effect on the slope factor *z_q_* of the *(1-ΔF/F)-V* distribution (∼0.26–0.27 with, versus ∼0.48–0.49 without extracellular Na^+^ ions). As outlined in Supporting Information (see **Appendix S2**, **Appendix S3**, and Figure S1), such a situation can arise from a superposition of effects resulting from cation binding through an intra- and an extracellular access channel. For the pseudo three-state model depicted in Fig. 1B, the following assumptions are made: First, the *z_q_* factor of ∼0.5 measured in the absence of external Na^+^ exclusively represents the fractional depth of an intracellular H^+^ access channel. Second, external Na^+^ ions exert their effect on the conformational distribution by binding through a shallower extracellular ion well with a *z_q_* of ∼0.2. This is reasonable, since the H,K-ATPase lacks the third ‘unique’ cation binding site characteristic for the Na^+^ pump, which is responsible for the major electrogenic release of the third Na^+^ ion with a fractional charge of ∼0.8, whereas the release/uptake of cations to the two ‘common’ sites occurs with a smaller apparent valence of ∼0.2. The model simulations in **Appendix S3** qualitatively reproduce the experimental observations (Figure S1): First, the inclusion of an additional electrogenic extracellular Na^+^ uptake step enforces a positive shift in *V_0.5_*. Second, such uneven *z_q_* factors distort the voltage dependence of the resultant conformational distribution in a way that fitting by a simple Boltzmann-type function yields an ‘effective’ *z_q_* value of even less than 0.5 (**Appendix S3** and Figure S1), exactly as observed in Fig. 5A.

Thus, the voltage dependence of the calculated rate constants *k_f_* and *k_b_* of the forward and backward reaction (Fig. 2E,F and Fig. 5D,E,G,H) can be reconciled with the concept of a high-field intracellular and a shallower extracellular access channel. Although more detailed kinetic information would be required to correlate the calculated rate constants *k_f_* and *k_b_* with individual rate constants within the pseudo three-state model of **Appendix S2**, a tentative assignment appears feasible. The data in Fig. 5D,E,G,H show that one of the rates, *k_b_*, is rather strongly dependent on membrane potential (with *z_q_* values of ∼0.23), whereas the voltage dependence of the other, *k_f_*, is very weak. In case of the Na^+^ pump, the voltage insensitivity of the forward rate constant from ouabain-sensitive transient currents is attributed to a voltage-independent reaction step (the E_1_P→E_2_P conformational transition in conjunction with Na^+^ deocclusion) that is rate-limiting the subsequent Na^+^ release step(s). The increase of rate constants upon hyperpolarization results from the fact that negative potentials favor the entry of Na^+^ ions to the binding pocket through an extracellular high-field access channel. With an hypothetical intracellular access channel in the case of H,K-ATPase, the fact that the forward rate constant *k_f_* is independent from voltage (and pH_ex_) suggests that a voltage-independent step preceding intracellular H^+^ binding (step 1 in Fig. 1A) is rate-limiting for H^+^ uptake and the subsequent E_1_P→E_2_P conformational transition. Conversely, the relatively steep voltage dependence of *k_b_* results from the fact that negative voltages speed up the intracellular release of H^+^ through the access channel. The [Na^+^]_ex_-dependence of the partial reaction represented by *k_b_* might be the consequence of Na^+^ ions traversing a shallow extracellular access channel to reach the binding pocket, which, in effect, will also speed up the E_2_P→E_1_P transition.

### Proposed mechanism for the effect of intracellular pH on the H^+^/K^+^ pumping rate

The dependence of the proposed voltage-independent step preceding intracellular H^+^ binding on the intracellular pH could mean that prior to the electrogenic binding of H^+^ to the transport site(s) a proton must bind to a ‘regulatory’ site (with a pK_a_ around neutral), which is in rapid equilibrium with the intracellular pH. Neutralization of a protonatable residue might change the local electrostatics, eventually leading to the formation of the access channel itself or to control the accessibility of the ion well for intracellular protons. Alternatively, the ‘regulatory’ proton could even be one of the presumably two protons that are transported in each reaction cycle. Of note, our Rb^+^ uptake experiments suggest that the availability of protons at the intracellular side is not only rate-limiting for the E_1_P→E_2_P conformational transition (Fig. 3G), but also for the turnover rate during stationary cation pumping (Fig. 3H). This conclusion can be drawn from the fact that both the stationary turnover number (Rb^+^ uptake) and the forward rate constant of the E_1_P↔E_2_P relaxation (monitored by the VCF experiments) show an about two-fold increase upon intracellular acidification by 0.5 pH units. Importantly, at pH_ex_ 7.4 as well as pH_ex_ 5.5, only a very small decrease of the Rb^+^ transport activity was observed at −100 mV compared to unclamped oocytes, whereas the about two-fold increase of Rb^+^ transport at pH_ex_ 5.5 compared to pH_ex_ 7.4 occurred irrespective of the membrane potential. The weak voltage sensitivity of cation transport is, first, reflected by the hardly voltage-sensitive rate constants *k_f_* (Figure 5D,E,G,H). Second, if the rate constant for K^+^(Rb^+^)-dependent dephosphorylation is much faster than the voltage-dependent relaxation between E_1_P and E_2_P, the majority of H,K-ATPase molecules on average will dwell in states (e.g. dephosphorylated intermediates like E_1_), whose occupancies are insensitive to transmembrane voltage, as indicated from the small voltage-dependent fluorescence changes under turnover conditions (Fig. 4).

The close similarity of the activation energies at pH_ex_ 7.4 and 5.5 also suggests that Rb^+^ uptake of the gastric proton pump is rate-limited by the same pH_in_-dependent partial reaction, and the high E_A_ values suggest that this step is likely not diffusion-controlled, but might be related to a major conformational change. The activation energies at pH_ex_ 7.4 and pH_ex_ 5.5 reported here are remarkably close to the 93 kJ/mol determined by Stengelin and co-workers in BLM experiments at an intermediate pH of 6.2. [21]. This value was obtained from the temperature-dependence of a time constant (τ_3_) that was assigned to the phosphorylation reaction and covered an even larger temperature range between 3°C and 40°C. The agreement between these values corroborates the idea that the overall pump activity monitored by the Rb^+^ uptake measurements is indeed rate-limited by partial reactions of the H^+^ outward moving branch, i.e. the phosphorylation reaction (that strongly depends on the intracellular H^+^ concentration) and the subsequent E_1_P→E_2_P conformational transition reflected by the presteady-state fluorescence measurements.

### Proposed role of Glu-820 for intra- and extracellular proton sensitivity

In a recent study, we have identified an acidic residue belonging to the putative cation binding pocket of the gastric H,K-ATPase (Glu-820 in M6) that might be crucial for the sensitivity towards intracellular acidification described here. Upon replacement of Glu-820 by non-protonatable residues (e.g. Gln or Ala), Rb^+^ uptake did not increase in presence of butyrate at pH_ex_ 7.4 (see Fig. 6F in [40]), which is very different from the aforementioned behavior of the wild-type enzyme. At pH_ex_ 5.5, Rb^+^ uptake by the two charge-neutralizing mutants was even reduced indicating that the mutations result in an increased competition of extracellular protons with Rb^+^ ions at the binding sites. Therefore, Glu-820 could be crucial for determining K^+^ (Rb^+^) selectivity in the E_2_P state, which is especially important at steep H^+^ gradients. Glu-820 may represent a site were protons are transiently bound before being expelled to the extracellular space, since the proximity of Glu-820 to the charged side-chain of Lys-791 (see Figure S2) could facilitate the large pK changes that are required to enable expulsion of a proton from this site at physiological pH of ∼1 into the stomach lumen. If the site is not occupied by a proton, the two oppositely charged residues Lys-791 and Glu-820 are probably forming a salt bridge that stabilizes the pump in the E_2_P state, as proposed earlier [40], [52].

### Concluding remarks

In the absence of extracellular K^+^, extracellular acidification from pH_ex_ 7.4 to 5.5 has no effect on the E_1_P↔E_2_P relaxation of gastric H,K-ATPase. In contrast, intracellular acidification by ∼0.5 pH units speeds up the forward relaxation rate and increases the H^+^/K^+^ pumping rate two-fold. Extracellular Na^+^ ions compete with protons and K^+^ ions for entry into the extracellular-facing access channel to the binding sites in E_2_P, but have no significant effect on the dephosphorylation branch of the cycle. Kinetic analysis based on a pseudo-three state model that simultaneously includes voltage-dependent (un)binding/(de)occlusion steps through an intra- and an extracellular access channel indicates that the intracellular access channel for protons has a fractional depth of ∼0.5, whereas the extracellular access channel, which is accessible for protons, Na^+^ and K^+^ ions, has a fractional depth of ∼0.2. The overall H^+^/K^+^ pumping rate is essentially voltage-insensitive indicating that a voltage-independent step is rate-limiting for the pump cycle. This intracellular pH-sensitive, rate-limiting step might be the intracellular binding of a proton to a regulatory binding site, which could be the transport site, to which the side chain of E820 is contributing.

## Supporting Information

Figure S1 **Model simulations.** (A) Simulation curves for the function from **Eq. B23** (see **Appendix S2**) with parameters *B* = 1, *z_qi_* = 0.5 and z*_qo_* = 0 for the fractional depth of the intra- or extracellular access channel, respectively. Variation of *A* alters the saturation value of *F*(*V*) and leads to a shift in *V_0.5_*. (B) Simulation curves for the function from **Eq. B23** with parameters *A* = 0.3, *z_qi_* = 0.5 and z*_qo_* = 0 for the fractional depth of the intra- or extracellular access channel, respectively. Variation of *B* shifts the *V_0.5_* value of the distribution in a logarithmic fashion. (C) Simulated data (dots) according to **Eq. B23** with parameters *A* = 2, B = 1, *z_qi_* = 0.5 and z*_qo_* values of 0 (•), 0.2 (•) and 0.5 (▪) for the fractional depth of the intra- or extracellular access channel, respectively. Also included are fits of a Boltzmann-type function to the simulated data sets (solid lines) with fit parameters as indicated.
(TIF)

Figure S2 **Structural model or rat gastric H,K-ATPase.** Structural model of the rat gastric H,K-ATPase according to PDB structure entry 3B8E (Morth et al. (2007), Nature **450**: 1043–1048; doi:10.1038/nature06419), which represents pig renal Na,K-ATPase in the E2•P_i_ conformation with two bound Rb^+^ ions. The structure model was created using SwissModel (http://swissmodel.expasy.org/) after manual adjustment of the sequence alignment according to the data deposited in The P-type ATPase Database (http://traplabs.dk/patbase/). The left panel shows an overview of the domain structure of H,K-ATPase with nucleotide binding (N), phosphorylation (P), actuator (A) and transmembrane (TM) domain indicated by different colors. Also shown is the transmembrane part of the β-subunit (light blue), the β-subunit's ectodomain, which was not resolved in the 3B8E structure, is omitted for clarity. Highlighted in red is the central β-sheet of the P domain close to D385, the residue, which is intermediately phosphorylated during the reaction cycle. Furthermore, two bound Rb^+^ ions are shown within the putative binding pocket in the center of the block of transmembrane helices, and the enzyme's C-terminus (dark blue) including the two terminal tyrosines, which have been shown to be pivotal for cation transport in Na,K-ATPase. Depicted in orange is the central transmembrane helix M5, whose upper part extends into the P domain, whereas in the TM region residue K791 is located, which contributes to cation coordination. Close to the extracellular end of M5 within the M5/M6 loop the Cys mutation S806C is shown, to which the fluorescent dye tetramethylrhodamine-maleimide (TMRM) is site-specifically bound. The right panel shows the transmembrane region in higher magnification using the same color coding as on the left. Here, the location of the putatively salt bridge-forming residues K791 (M5) and E820 (M6) in the vicinity of the bound Rb^+^ ions is shown in relation to the labeling position S806C, which resides at the extracellular mouth of the cation exit pathway.
(TIF)

Appendix S1 Simplified two-state kinetic model used for analysis of voltage-dependent fluorescence signals.
(DOC)

Appendix S2 Pseudo three-state model including charge translocation through intra- and extracellular-facing access channels used for model simulations to rationalize experimental observations.
(DOC)

Appendix S3 Model simulations to elucidate the impact of voltage-dependent parameters and rate constants on the conformational distribution of H,K-ATPase.
(DOC)
