# Supplementary figures and images for: Control of Gastric H,K-ATPase Activity by Cations, Voltage and Intracellular pH Analyzed by Voltage Clamp Fluorometry in Xenopus Oocytes
Source: PLoS One. 2012 Mar 20;7(3):e33645. doi: 10.1371/journal.pone.0033645 (PMC3308979; doi:10.1371/journal.pone.0033645)

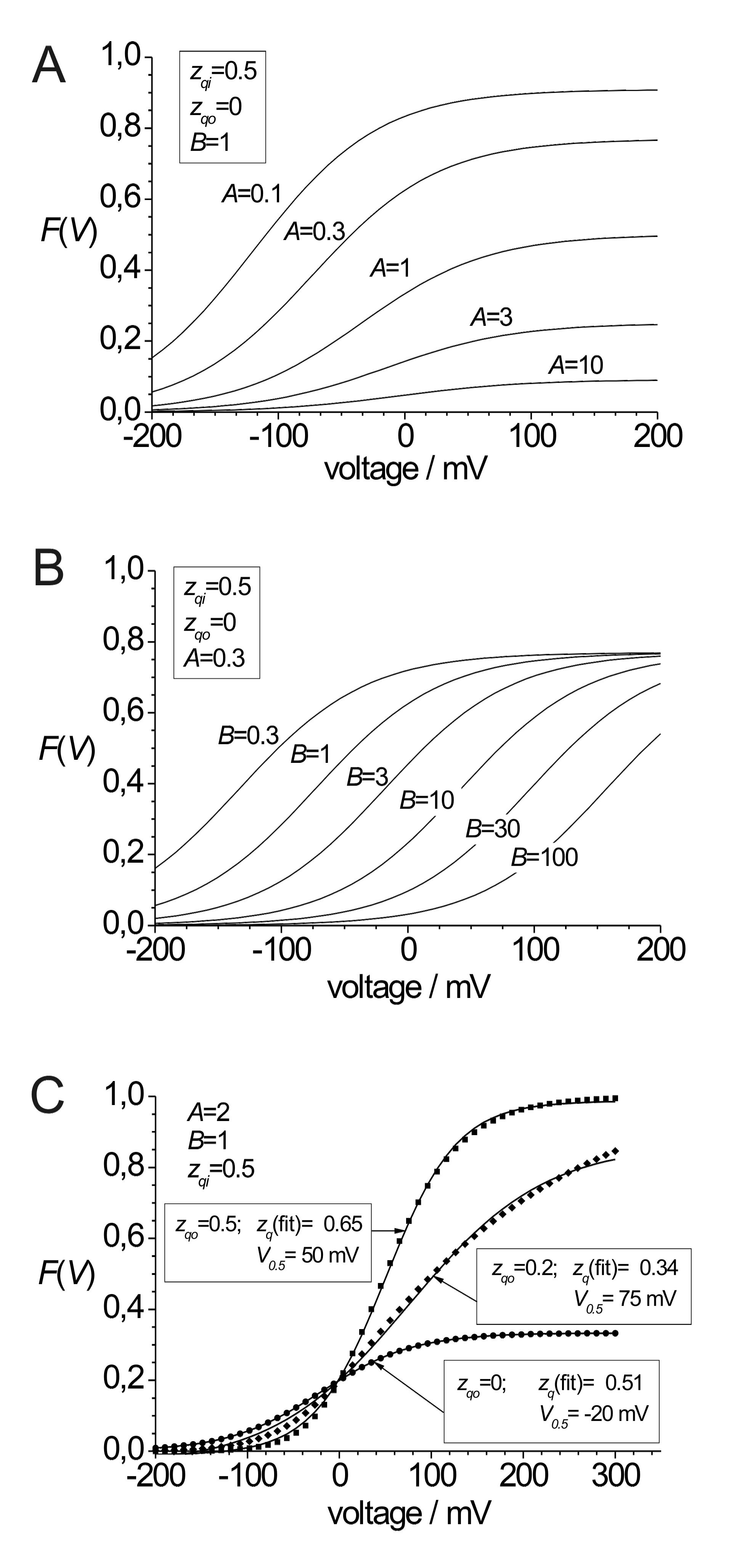

Supplement: Figure S1 — Model simulations. (A) Simulation curves for the function from Eq. B23 (see Appendix S2) with parameters B = 1, zqi = 0.5 and zqo = 0 for the fractional depth of the intra- or extracellular access channel, respectively. Variation of A alters the saturation value of F(V) and leads to a shift in V0.5. (B) Simulation curves for the function from Eq. B23 with parameters A = 0.3, zqi = 0.5 and zqo = 0 for the fractional depth of the intra- or extracellular access channel, respectively. Variation of B shifts the V0.5 value of the distribution in a logarithmic fashion. (C) Simulated data (dots) according to Eq. B23 with parameters A = 2, B = 1, zqi = 0.5 and zqo values of 0 (•), 0.2 (•) and 0.5 (▪) for the fractional depth of the intra- or extracellular access channel, respectively. Also included are fits of a Boltzmann-type function to the simulated data sets (solid lines) with fit parameters as indicated. (TIF) [file pone.0033645.s001.tif]

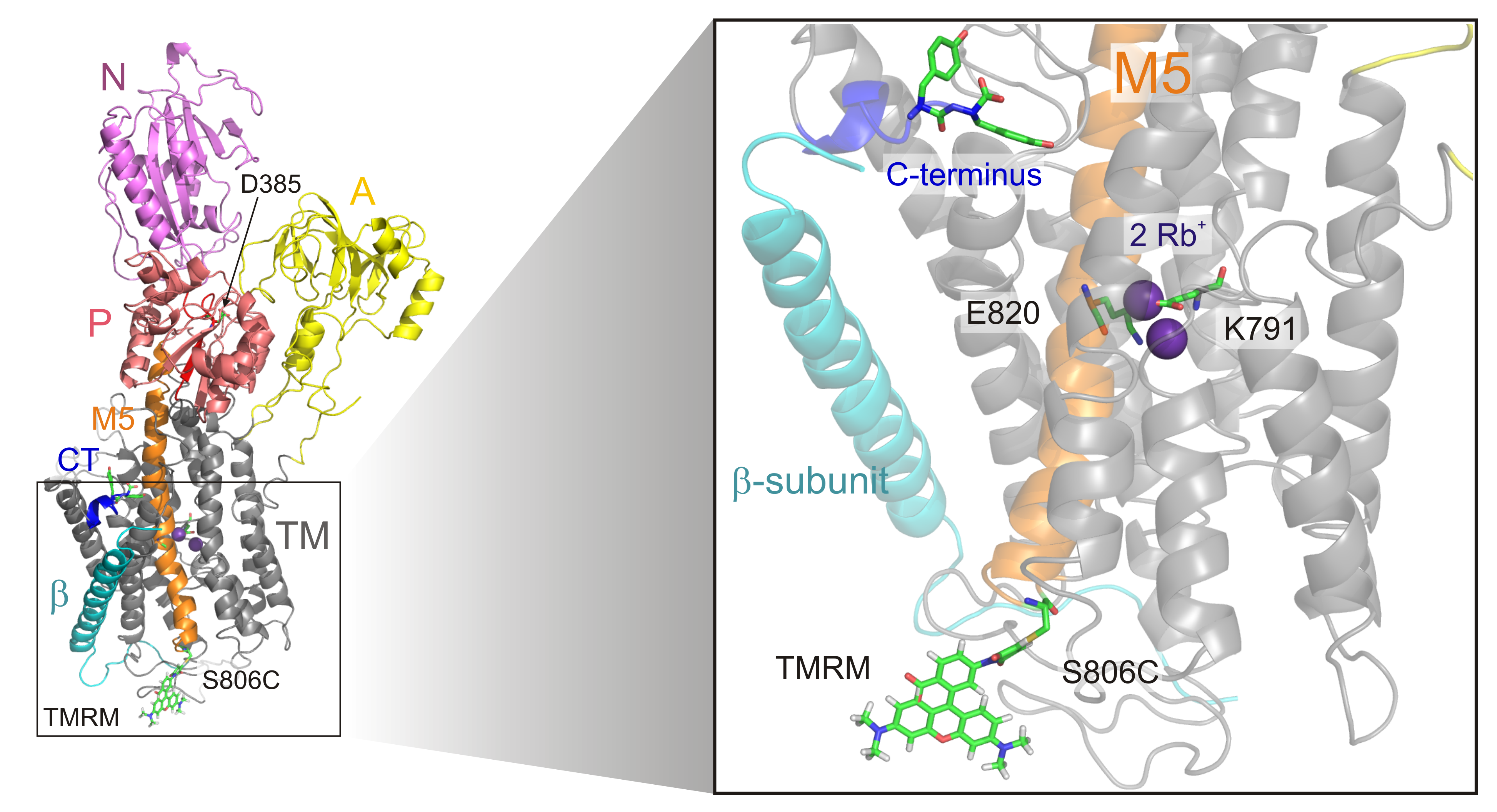

Supplement: Figure S2 — Structural model or rat gastric H,K-ATPase. Structural model of the rat gastric H,K-ATPase according to PDB structure entry 3B8E (Morth et al. (2007), Nature 450: 1043–1048; doi:10.1038/nature06419), which represents pig renal Na,K-ATPase in the E2•Pi conformation with two bound Rb+ ions. The structure model was created using SwissModel (http://swissmodel.expasy.org/) after manual adjustment of the sequence alignment according to the data deposited in The P-type ATPase Database (http://traplabs.dk/patbase/). The left panel shows an overview of the domain structure of H,K-ATPase with nucleotide binding (N), phosphorylation (P), actuator (A) and transmembrane (TM) domain indicated by different colors. Also shown is the transmembrane part of the β-subunit (light blue), the β-subunit's ectodomain, which was not resolved in the 3B8E structure, is omitted for clarity. Highlighted in red is the central β-sheet of the P domain close to D385, the residue, which is intermediately phosphorylated during the reaction cycle. Furthermore, two bound Rb+ ions are shown within the putative binding pocket in the center of the block of transmembrane helices, and the enzyme's C-terminus (dark blue) including the two terminal tyrosines, which have been shown to be pivotal for cation transport in Na,K-ATPase. Depicted in orange is the central transmembrane helix M5, whose upper part extends into the P domain, whereas in the TM region residue K791 is located, which contributes to cation coordination. Close to the extracellular end of M5 within the M5/M6 loop the Cys mutation S806C is shown, to which the fluorescent dye tetramethylrhodamine-maleimide (TMRM) is site-specifically bound. The right panel shows the transmembrane region in higher magnification using the same color coding as on the left. Here, the location of the putatively salt bridge-forming residues K791 (M5) and E820 (M6) in the vicinity of the bound Rb+ ions is shown in relation to the labeling position S806C, which resides a [file pone.0033645.s002.tif]
