# Supplementary material for: Control of Gastric H,K-ATPase Activity by Cations, Voltage and Intracellular pH Analyzed by Voltage Clamp Fluorometry in Xenopus Oocytes
Source: PLoS One. 2012 Mar 20;7(3):e33645. doi: 10.1371/journal.pone.0033645 (PMC3308979; doi:10.1371/journal.pone.0033645)
Supplement: Appendix S1 — Simplified two-state kinetic model used for analysis of voltage-dependent fluorescence signals. (DOC) [file pone.0033645.s003.doc]

**Appendix S1: Two-state kinetic model for data analysis**

A simplified two-state kinetic model was employed for the kinetic analysis of the voltage-dependent fluorescence changes observed with TMRM-labeled H,K-ATPase, based on reversible transition between the phosphointermediates E_1_P and E_2_P:

The E_1_P state is formed from a rapid pre-equilibrium reaction involving intracellular H^+^ binding and phosphorylation from ATP, whereas E_2_P is in rapid pre-equilibrium with an extracellular cation uptake/release. In analogy to previous findings on TMRM-labeled Na,K-ATPase [1], we assume that the voltage jump-induced fluorescence changes of TMRM-labelled H,K-ATPase reflect the voltage-dependent redistribution of enzyme between E_1_P and E_2_P states, whereby E_1_P or E_2_P are characterized by high or low fluorescence, respectively.

For simplification, we assume that the voltage-dependence of the forward and reverse rate constant (*k_f_* and *k_b_*) can be described by a single exponential function.

# $k_{f}(V)=k_{f}(0)\cdot e^{\frac{z_{q,f\cdot}\cdot F\cdot V}{RT}}$ Equation A1

# $k_{b}(V)=k_{b}(0)\cdot e^{-\left(\frac{z_{q,b\cdot}\cdot F\cdot V}{RT}\right)}$ Equation A2

# *k_f_*(0) and *k_b_*(0) are the respective values of the rate constants at 0 mV.

As outlined in **Appendix S2**, voltage dependence of *k_b_* can either result from a voltage-dependent first order rate constant for proton release through an intracellular ion well (**Eq. B16** in **Appendix S2**) or from a voltage-dependent pseudo-first order rate constant (**Eq. B4** in **Appendix S2**), which reflects the effect of the membrane voltage altering the ‘effective’ cation concentration within an extracellular access channel. Voltage dependence of *k_f_* can result from either a voltage-dependent first order rate constant for cation release through an extracellular ion well (**Eq. B3** in **Appendix S2**) or from a voltage-dependent pseudo-first order rate constant, which reflects the effect of the membrane voltage altering the ‘effective’ proton concentration within an intracellular access channel (**Eq. B17** in **Appendix S2**).

At a given holding potential *V_h_*, the fluorescence signals start at a certain level determined by the poise of the E_1_P/E_2_P distribution at *V_h_*. Upon a change in membrane potential to *V*, the system relaxes to the new equilibrium with a reciprocal time constant $\tau^{-1}(V)=k_{tot}(V)$, which is equal to the sum of the forward and backward rate constant at the potential *V*.

$k_{tot}(V)=k_{f}(V)+k_{b}(V)$ **Equation A3**

After background subtraction and normalization, the fluorescence amplitudes (*(1-ΔF/F)-V* values) are identified with a voltage-dependent fluorescence amplitude *F_L_(V)*, which indicates the fractional amount [*E_2_P*] of the E_2_P intermediate (with [*E_1_P*]+[*E_2_P*]=1). The amount of E_2_P is determined by the following ratio:

$F_{L}(V)=[E_{^{2}}P]=\frac{k_{f}(V)}{k_{f}(V)+k_{b}(V)}$ **Equation A4**

Insertion of **Eq. A1** and **Eq. A2** into **Eq. A4** yields an expression for the voltage dependence of fluorescence amplitudes *F_L_*(*V*), which is equivalent to a Boltzmann-type function:

$$F_{L}(V)=\frac{k_{f}(V)}{k_{f}(V)+k_{b}(V)}=\frac{k_{f}(0)\cdot e^{\frac{z_{q,f}\cdot F\cdot V}{RT}}}{k_{f}(0)\cdot e^{\frac{z_{q,f\cdot}\cdot F\cdot V}{RT}}+k_{b}(0)\cdot e^{-\left(\frac{z_{q,b\cdot}\cdot F\cdot V}{RT}\right)}}=\frac{1}{1+\frac{k_{b}(0)}{k_{f}(0)}\cdot e^{-\left(\frac{(z_{q,f\cdot}+z_{q,b})\cdot F\cdot V}{RT}\right)}}$$

with $z_{q(tot)}=z_{q,f}+z_{q,b}$

$F_{L}(V)=\frac{1}{1+\frac{k_{b}(0)}{k_{f}(0)}\cdot e^{-\left(\frac{(z_{q(tot)})\cdot F\cdot V}{RT}\right)}}$ **Equation A5**

By rearrangement of **Eq. A3** and **Eq. A4**, the individual voltage-dependent rate constants *k_f_(V)* and *k_b_(V)* can be calculated from the voltage-dependence of the total observed reciprocal time constants *k_tot_(V)* and the fluorescence amplitudes *F_L_(V)*:

$k_{f}(V)=F_{L}(V)\cdot k_{tot}(V)=F_{L}(V)\cdot\tau^{-1}(V)$ **Equation A6**

$k_{b}(V)=k_{tot}(V)\cdot\left(1-F_{L}(V)\right)=\tau^{-1}(V)\cdot\left(1-F_{L}(V)\right)$ **Equation A7**

Fitting the calculated voltage-dependent values for the forward rate constant (*k_f_*(*V*)*,* plotted as triangles in Figure 2 E,F and Figure 5 D,E,G,H) by a single exponential (**Eq. A1**) and those for the reverse rate constant (*k_b_*(*V*), inverted triangles in Figure 2 E,F and Figure 5 D,E,G,H) by **Eq. A2** yields the slope factors for the forward and reverse rate constants, *z_q,f_* and *z_q,b_*. In Table 1 the resulting values for *z_q,f_* and *z_q,b_* are listed together with the *z_q_* values, which had been obtained by fitting a Boltzmann-type equation (**Eq. A5**) to the voltage-dependent fluorescence amplitudes (*(1-ΔF/F)*-V distributions shown in Figure 2C and Figure 5 A,B).

**Reference cited**

1. Geibel S, Kaplan JH, Bamberg E, Friedrich T (2003) Conformational dynamics of the Na^+^/K^+^-ATPase probed by voltage clamp fluorometry. Proc Natl Acad Sci U S A 100: 964-969.
