# Supplementary material for: Control of Gastric H,K-ATPase Activity by Cations, Voltage and Intracellular pH Analyzed by Voltage Clamp Fluorometry in Xenopus Oocytes
Source: PLoS One. 2012 Mar 20;7(3):e33645. doi: 10.1371/journal.pone.0033645 (PMC3308979; doi:10.1371/journal.pone.0033645)
Supplement: Appendix S2 — Pseudo three-state model including charge translocation through intra- and extracellular-facing access channels used for model simulations to rationalize experimental observations. (DOC) [file pone.0033645.s004.doc]

**Appendix S2: Pseudo three-state model**

The H^+^-translocating branch of the H,K-ATPase reaction cycle is expressed in terms of a pseudo three-state model (Fig. 1B), which includes charge translocation through intra- and extracellular-facing access channels. For simplicity, the pseudo two-state scheme underlayed in gray (Fig. 1B) is considered first, which expresses the charge translocation in the presence of an extracellular-facing high-field access channel according to [1], which followed a previous description by Läuger and Apell [2]. In this scheme, *k_2_* represents the first-order rate constant for the transition from the *E_1_P*(*S^+^*) state with occluded cations (*S^+^*) to a state with extracellular-facing binding sites and cations released (*E_2_P*). *k_-2_* is the pseudo-first order rate constant for the reverse reaction, which is given as the product of a second-order rate constant *k_-2_*(0) (“0” denotes 0 mV membrane potential) and the effective cation concentration at the extracellular-facing binding sites.

The time course for the equilibrium distribution of cations between the bulk and the binding site is assumed to be rapid. Since cations bind to and are released from *E_2_P*, the time course of *E_2_P* is a measure of the conformational distribution, with [*E_1_P*(*S^+^*)]+[*E_2_P*]=1. Upon perturbation of the equilibrium between *E_1_P*(*S^+^*) and *E_2_P*, the system would relax to a new equilibrium value with a rate constant given as the sum of *k_2_* and *k_-2_*, and the system of differential equations yields the following solution for the time course of [*E_2_P*]:

$[E_{2}P](t)=\frac{k_{2}}{k_{2}+k_{-2}}\cdot\left[1-e^{-(k_{2}+k_{-2})\cdot t}\right]$ **Equation B1**

The steady-state solution (for $t\to\infty$) is given by:

$[E_{2}P]_{(t\to\infty)}=\frac{k_{2}}{k_{2}+k_{-2}}=\frac{1}{1+\frac{k_{-2}}{k_{2}}}$ **Equation B2**

If an extracellular access channel is assumed, both, *k_2_* and *k_-2_*, could be voltage-dependent. Since positive voltages should favor extracellular cation release, *k_2_* would have to include a Boltzmann factor that increases with depolarizing potentials:

$k_{2}=k_{2}(0)\cdot e^{z_{qo}\cdot\frac{F\cdot V}{R\cdot T}}$ **Equation B3**

In this expression, *k_2_*(0) is the rate constant at 0 mV and *z_q_* the fractional depth (equivalent charge) of the access channel for the release reaction. Voltage dependence of *k_-2_* arises as the consequence of the membrane voltage altering the ‘effective’ cation concentration (as opposed to the concentration in the external bulk solution [*S*^+^]*_o_*) at the binding site within the access channel of fractional depth *z_qo_* according to a Boltzmann equilibrium:

$k_{-2}=k_{-2}(0)\cdot[S^{+}]_{o}\cdot e^{-z_{qo}\cdot\frac{F\cdot V}{R\cdot T}}$ **Equation B4**

*k_-2_* increases with hyperpolarization, since negative potentials increase the effective cation concentration within the extracellular access channel. *k_‑2_*(0) denotes the value at 0 mV.

If either only *k_-2_* is assumed to be voltage-dependent (i.e. $k_{2}=const.=k_{2}(0)$), or only *k_2_* (i.e. $k_{-2}=const.=k_{-2}(0)\cdot[S^{+}]_{o}$), substituting **Eq. B3** or **Eq. B4** into **Eq. B2** yield the same expression:

$[E_{2}P]_{(t\to\infty)}=\frac{1}{1+\frac{k_{-2}(0)\cdot[S^{+}]_{o}}{k_{2}(0)}\cdot e^{-z_{qo}\cdot\frac{F\cdot V}{R\cdot T}}}$ **Equation B5**

This equation describes a voltage-dependent, sigmoidal, Boltzmann-type function with a slope factor *z_qo_* and a midpoint potential *V_0.5_* , which is defined as the potential at which $[E_{2}P]_{(t\to\infty)}$ approaches 0.5 such that

$\frac{k_{-2}(0)\cdot[S^{+}]_{o}}{k_{2}(0)}=e^{z_{qo}\cdot\frac{F\cdot V_{0.5}}{R\cdot T}}$ **Equation B6**

Substituting **Eq. B6** into **Eq. B5** yields:

$[E_{2}P]_{(t\to\infty)}=\frac{1}{1+e^{-z_{qo}\cdot\frac{F\cdot(V-V_{0.5})}{R\cdot T}}}$ **Equation B7**

Rearrangement of **Eq. B6** gives an expression for *V_0.5_*:

$V_{0.5}=\frac{R\cdot T}{z_{qo}\cdot F}\ln\left[\frac{k_{-2}(0)\cdot[S^{+}]_{o}}{k_{2}(0)}\right]$ **Equation B8**

Thus, a change in [*S^+^*]*_o_* from [*S^+^*]*_I_* to [*S^+^*]*_II_* leads to a shift in *V_0.5_* by

$\Delta V_{0.5}=\frac{R\cdot T}{z_{qo}\cdot F}\ln\frac{[S^{+}]_{II}}{[S^{+}]_{I}}$ **Equation B9**

which is given as *Equation 1* in the main text of the article.

If the general case is considered that *k_2_* and *k_-2_* are both voltage-dependent, we set *z_2f_* in **Eq. B3** and *z_2b_* in **Eq. B4** as the apparent valences for the forward and backward reaction, respectively, and insert these expressions into **Eq. B2**, which yields:

$[E_{2}P]_{(t\to\infty)}=\frac{1}{1+\frac{k_{-2}}{k_{2}}}=\frac{1}{1+\frac{k_{-2}(0)\cdot[S^{+}]_{o}}{k_{2}(0)}\cdot e^{-(z_{2b}+z_{2f})\cdot\frac{F\cdot V}{R\cdot T}}}$ **Equation B10**

Like **Eq. B5**, this describes a Boltzmann-type function with a slope factor given by the sum *z_2b+_ z_2f_* and a midpoint potential *V_0.5_* as follows:

$V_{0.5}=\frac{R\cdot T}{(z_{2b}+z_{2f})\cdot F}\cdot\ln\left[\frac{k_{-2}(0)\cdot[S^{+}]_{o}}{k_{2}(0)}\right]$ **Equation B11**

Thus, without any further kinetic information, a sigmoidally voltage-dependent conformational distribution according to **Eq. B5** or **Eq. B11** can be due to voltage dependence of *k_2_*, *k_-2_*, or both, including also the ‘asymmetric’ case of unequal equivalent charges for the forward and backward reaction. Therefore, the determination of the individual rate constants is required to assign voltage dependence to specific reaction steps.

In case of the Na,K-ATPase, the reciprocal time constants from voltage jump-induced transient currents increase with hyperpolarization and show a constant plateau at positive potentials. It has been concluded from these observations that only the reverse binding of Na^+^ through the external-facing access channel is voltage dependent, whereas Na^+^ release is rate-limited (thus voltage-independent) by the preceding slow and voltage-independent conformational transition/occlusion reaction.

Next, also a cation uptake or release reaction through an intracellular-facing access channel by a transition between the *E_1_P*(*S^+^*) state with occluded cations (*S^+^*) and a state with intracellular-facing binding sites (*E_1_P*) shall be included (pseudo three-state reaction scheme in Fig. 1B). *k_2_* and *k_-2_* are defined as above, with *z_qo_* representing the apparent valence (fractional depth) of the extracellular access channel. Moreover, *k_-1_* denotes the first-order rate constant for intracellular cation deocclusion/release. *k_1_* is a pseudo-first order rate constant for the intracellular cation binding reaction, which is given as the product of a second-order rate constant *k_1_*(0) and the effective cation concentration at the intracellular-facing binding sites, and *z_qi_* is the apparent valence (fractional depth) of the intracellular access channel.

The total amount of enzyme states is defined as:

$[E_{1}P]+[EP(S^{+})]+[E_{2}P]=1$ **Equation B12**

The solution of the system of differential equations for the pseudo three-state scheme yields the following solution for [*E_2_P*] [2]:

$[E_{2}P](t)=C_{3}\cdot\left[\frac{1}{a\cdot b}+\frac{b\cdot e^{-at}-a\cdot e^{-bt}}{a\cdot b\cdot(a+b)}\right]$ **Equation B13**

with: $C_{1}=k_{1}+k_{-1}+k_{2}+k_{-2}$

$$C_{2}=k_{1}\cdot k_{2}+k_{1}\cdot k_{-2}+k_{-1}\cdot k_{-2}$$

$$C_{3}=k_{1}\cdot k_{2}$$

$$a=\frac{C_{1}+\sqrt{C_{1}^{2}-4C_{2}}}{2}$$

$$b=\frac{C_{1}-\sqrt{C_{1}^{2}-4C_{2}}}{2}$$

Of note, $a\cdot b=C_{2}$. The steady-state solution (for $t\to\infty$) is given by:

**Equation B14**

First, we consider that only extracellular release or reverse binding of cations through an access channel with fractional depth *z_qo_* is electrogenic (*k_1_* and *k_-1_* are voltage-independent). If either only *k_-2_* is assumed to be voltage-dependent (i.e. $k_{2}=const.=k_{2}(0)$), or only *k_2_* (i.e. $k_{-2}=const.=k_{-2}(0)\cdot[S^{+}]_{o}$), substituting **Eq. B3** or **Eq. B4** into **Eq. B14** yields:

$[E_{2}P]_{(t\to\infty)}=\frac{1}{1+\left[1+\frac{k_{-1}}{k_{1}}\right]\cdot\frac{k_{-2}(0)}{k_{2}(0)}\cdot[S^{+}]_{o}\cdot e^{-z_{qo}\cdot\frac{F\cdot V}{R\cdot T}}}$ **Equation B15**

This equation, again, describes a Boltzmann-type function with a slope factor *z_qo_* and a midpoint potential *V_0.5_* defined by the condition:

$\left[1+\frac{k_{-1}}{k_{1}}\right]\cdot\frac{k_{-2}(0)}{k_{2}(0)}\cdot[S^{+}]_{o}=e^{z_{qo}\cdot\frac{F\cdot V_{0.5}}{R\cdot T}}$ **Equation B16**

**Eq. B16** can be rearranged to give an explicit expression of *V_0.5_* :

$V_{0.5}=\frac{R\cdot T}{z_{qo}\cdot F}\cdot\ln\left[\frac{(k_{1}+k_{-1})\cdot k_{-2}(0)\cdot[S^{+}]_{o}}{k_{1}\cdot k_{2}(0)}\right]$ **Equation B17**

With this expression, **Eq. B15** can be written as follows, yielding essentially the same result as obtained from a pseudo two-state scheme (**Eq. B7**):

$[E_{2}P]_{(t\to\infty)}=\frac{1}{1+e^{-z_{qo}\cdot\frac{F\cdot(V-V_{0.5})}{R\cdot T}}}$ **Equation B18**

Second, we consider that only an intracellular ion well with fractional depth *z_qi_* is present (with voltage-independent *k_2_* and *k_-2_*). *k_-1_* now includes a Boltzmann factor that increases with hyperpolarization, since negative potentials favor intracellular cation release:

$k_{-1}=k_{-1}(0)\cdot e^{z_{qi}\cdot\frac{F\cdot V}{R\cdot T}}$ **Equation B19**

Voltage dependence of the pseudo-first order rate constant *k_1_* arises from the assumption that positive membrane voltages increase the ‘effective’ cation concentration (as opposed to the concentration in the intracellular solution, [*S*^+^]*_i_*) at the binding site within the intracellular access channel:

$k_{1}=k_{1}(0)\cdot[S^{+}]_{i}\cdot e^{z_{qi}\cdot\frac{F\cdot V}{R\cdot T}}$ **Equation B20**

If either only *k_1_* is assumed to be voltage-dependent (i.e. $k_{-1}=const.=k_{-1}(0)$), or only *k_-1_* (i.e. $k_{1}=const.=k_{1}(0)\cdot[S^{+}]_{i}$), insertion of **Eq. B18** or **Eq. B19** into **Eq. B14** yields:

$[E_{2}P]_{(t\to\infty)}=\frac{1}{1+\frac{k_{-2}}{k_{2}}+\frac{k_{-2}}{k_{2}}\cdot\frac{k_{-1}(0)}{k_{1}(0)\cdot[S^{+}]_{i}}\cdot e^{-z_{qi}\cdot\frac{F\cdot V}{R\cdot T}}}$ **Equation B21**

This equation also describes a Boltzmann-type function with a slope factor *z_qi_* and a midpoint potential *V_0.5_* , which can be defined by a condition as above. However, this distribution only assumes values between zero and $\frac{k_{2}}{k_{2}+k_{-2}}<1$.

A more complex situation results, when voltage-dependent cation binding or release reactions within an extra- *and* an intracellular ion well occur simultaneously. In this general case, insertion of **Eq. B3** (or **Eq. B4**) and **Eq. B19** (or **Eq. B20**) into **Eq. B14** yields:

**Equation B22**

This formula no longer describes a simple Boltzmann-type function with a well-defined slope factor, and the “*V_0.5_* value”, for which the distribution approaches 0.5, is an implicit function of the set of parameters.

**References cited**

1. Rakowski RF (1993) Charge movement by the Na/K pump in *Xenopus* oocytes. J Gen Physiol 101: 117-144.

2. Läuger P, Apell HJ (1988) Transient behaviour of the Na^+^/K^+^-pump: microscopic analysis of nonstationary ion-translocation. Biochim Biophys Acta 944: 451-464
