# Supplementary material for: Control of Gastric H,K-ATPase Activity by Cations, Voltage and Intracellular pH Analyzed by Voltage Clamp Fluorometry in Xenopus Oocytes
Source: PLoS One. 2012 Mar 20;7(3):e33645. doi: 10.1371/journal.pone.0033645 (PMC3308979; doi:10.1371/journal.pone.0033645)
Supplement: Appendix S3 — Model simulations to elucidate the impact of voltage-dependent parameters and rate constants on the conformational distribution of H,K-ATPase. (DOC) [file pone.0033645.s005.doc]

**Appendix S3: Model simulations**

To analyze qualitatively, how the various parameters affect the shape of the distribution expressed by **Eq. B22** (**Appendix S2**), we define new parameters *A* and *B* as follows

$A=\frac{k_{-2}(0)\cdot[S^{+}]_{o}}{k_{2}(0)}$, $B=\frac{k_{-1}(0)}{k_{1}(0)\cdot[S^{+}]_{i}}$ and use$\frac{F}{R\cdot T}=$25 mV

This results in a voltage-dependent function *F*(*V*) of the form:

$F(V)=\frac{1}{1+A\cdot\left[e^{-z_{qo}\cdot\frac{V[mV]}{25 mV}}+B\cdot e^{-(z_{qi}+z_{qo})\cdot\frac{V[mV]}{25 mV}}\right]}$ **Equation B23**

First, we consider the situation of the H,K-ATPase at an extracellular pH of 7.4. Since the pump releases protons against a luminal [H^+^] of about 150 mM, a pK_a_ value of below 1 has to be assumed for the extracellular-facing H^+^ binding sites. Thus, either the extracellular proton concentration is insufficient to achieve a critical increase the ‘effective’ H^+^ concentration at the binding site in a voltage-dependent fashion, or the extracellular ion well has to be considered as very shallow. Therefore, we set *z_qo_*=0 to eliminate the electrogenicity of the extracellular reverse binding reaction, choose *z_qi_*=0.5 (according to the slope of the conformational distribution in the absence of extracellular Na^+^, see Fig. 5A,B of the article), and assume parameter *B* to be constant (symbolizing constant intracellular [H^+^]). **Eq. B23** then yields:

$$F(V)=\frac{1}{1+A+A\cdot B\cdot e^{-z_{qi}\cdot\frac{V[mV]}{25 mV}}}$$

This expression is a Boltzmann-type function (equivalent to the situation described by **Eq. B21** in **Appendix S2**) with a slope factor determined by the fractional depth of the intracellular access channel. Of note, at a fixed value of *B*, this distribution only assumes values between 0 and $\frac{1}{1+A}$, and the *V_0.5_* value is dependent on *A*, as exemplified in Supporting Figure S1A.

Next, at a fixed *A* value of 0.3 (which, together with *B*=1, is chosen since the corresponding curve in Supporting Figure S1B is similar to the *(1-ΔF/F)-V* distribution at pH_ex_ 7.4 in the absence of Na^+^ (Fig. 5A of the article), we analyze the effect of changes in the intracellular proton concentration, which will affect the value of parameter *B*. An increase of [H^+^]_i_ will decrease *B* and shift the distribution towards negative potentials (Supporting Figure S1B).

The negative *V_0.5_* shift with increasing [H^+^]_in_ (decreasing *B*) is in agreement with the data presented in Fig. 3F of the article.

Finally, we consider the situation in the presence of high [Na^+^]_ex_. In this case, we assume that Na^+^ produces sufficient competition with H^+^ for the external-facing binding sites, such that *z_qo_* can no longer be neglected. Since the H,K-ATPase lacks the third ‘unique’ cation binding site characteristic for the Na^+^ pump, which is responsible for the major electrogenic release of the third Na^+^ ion with a fractional charge of ~0.8, whereas the release/uptake of cations to the two ‘common’ sites occurs with a smaller apparent valence of ~0.2, we set *z_qo_*=0.2. Furthermore, we set *B*=0.3 as before and use a slightly increased value *A*=2 to reflect the increased ‘effective’ cation concentration on the extracellular side (condition 1). In addition, we consider the case (condition 2) of ‘symmetric’ intra- and extracellular access channels with apparent valences *z_qo_* and *z_qi_* both set to 0.5, and compare it to the case (condition 3) with *z_qo_*=0. For the simulated curves for these three cases (Supporting Figure S1C), we also carried out fits of a simple Boltzmann-type function (**Eq. B7** in **Appendix S2**) to indicate, how the inclusion of a second electrogenic step from the opposite side of the membrane affects the ‘effective’ value of the fit parameter *z_q_*.

The simulations in Supporting Figure S1C show that the curve resulting from *z_qo_*=0 can well be approximated by a Boltzmann-type function with a fit parameter *z_q_* (0.51) that closely reflects the chosen *z_qi_* value of 0.5. With non-zero *z_qo_*, the distribution is no longer limited to values between zero and $\frac{1}{1+A}$, but assumes all values between zero and 1. In addition, the *V_0.5_* value is shifted to positive potentials, in line with the *V_0.5_* shift obtained upon addition of external Na^+^ as depicted in Fig. 5A of the article. Furthermore, the inclusion of a second electrogenic step on the opposite side of the membrane leads to a distortion of the *F*(*V*) distribution, which results in a decreased slope. With *z_qo_*=*z_qi_*=0.5, the ‘effective’ slope obtained from the fit (0.65) is smaller than the sum of both apparent valences. This effect is especially pronounced, if *z_qo_* is smaller than *z_qi_* (but not in the opposite case, data not shown), and with *z_qo_*=0.2 the ‘effective’ slope from the fit (0.34) is even smaller than *z_qi_*. These simulations show that the inclusion of an extracellular Na^+^-dependent electrogenic step with apparent valence 0.2 (representing a shallow extracellular ion well) can lead to a decrease in the ‘effective’ *z_q_* to a value that is smaller than the one obtained in the absence of extracellular Na^+^, in accordance with the observations shown in Fig. 5A of the article.
